# Lipid metabolism in homeostasis and disease

**DOI:** 10.1038/s41392-025-02357-x

**Published:** 2026-02-16

**Authors:** Zhenghao Li, Wende Deng, Lanxuan Yang, Changheng Tang, Jian-Min Yue, Olivia Monteiro, Daniel T. Baptista-Hon, Ting Li

**Affiliations:** 1 State Key Laboratory of Quality Research in Chinese Medicine & Faculty of Chinese Medicine, Macau University of Science and Technology, Macau SAR, China; 2 State Key Laboratory of Quality Research in Chinese Medicines & School of Pharmacy, Faculty of Medicine, Macau University of Science and Technology, Macau SAR, China; 3 State Key Laboratory of Drug Research, Shanghai Institute of Materia Medica, Chinese Academy of Sciences, Shanghai, China; 4 Faculty of Medicine, Macau University of Science and Technology, Macau SAR, China; 5 Macau Institute for Artificial Intelligence in Medicine, Macau University of Science and Technology, Macau SAR, China; 6 School of Medicine, University of Dundee, Dundee, UK; 7 Macau Institute for Applied Research in Medicine and Health, Macau University of Science and Technology, Macau SAR, China

**Keywords:** Inflammation, Immunological disorders

## Abstract

Lipid metabolism is essential for maintaining cellular homeostasis, and its dysregulation is linked to various diseases, including cancer, cardiovascular disease, and diabetes. Immune cells, such as macrophages, T cells, B cells, and neutrophils, rely on lipid metabolism for their function, which impacts both innate and adaptive immune responses. Understanding how lipid metabolism influences immune cells is crucial, as it can reveal new therapeutic opportunities for immune-mediated diseases. In this review, we provide a retrospective summary of the research history and milestone events in lipid metabolism research and highlight the importance of lipid metabolism in immune cells. In addition to discussing the various lipid functions, transport, and signaling pathways involved in lipid metabolism, we mainly explore the regulation of immune cell behavior by lipid metabolism, focusing on the roles of lipid metabolites in immune cell proliferation, differentiation, and activation. We further highlight multilevel regulatory mechanisms, including genetic, epigenetic, posttranscriptional, and posttranslational regulation, and their impact on immune cell function. Additionally, we discuss the role of lipid metabolism in diseases such as autoimmune diseases, cancer, neurodegenerative diseases, cardiovascular diseases, aging, and metabolic disorders. Finally, we summarize therapeutic strategies targeting lipid metabolism, the progress of global clinical trials, and future research directions, including lipid-derived biomarkers and innovative therapeutic approaches.

## Introduction

**Fig. 1:**
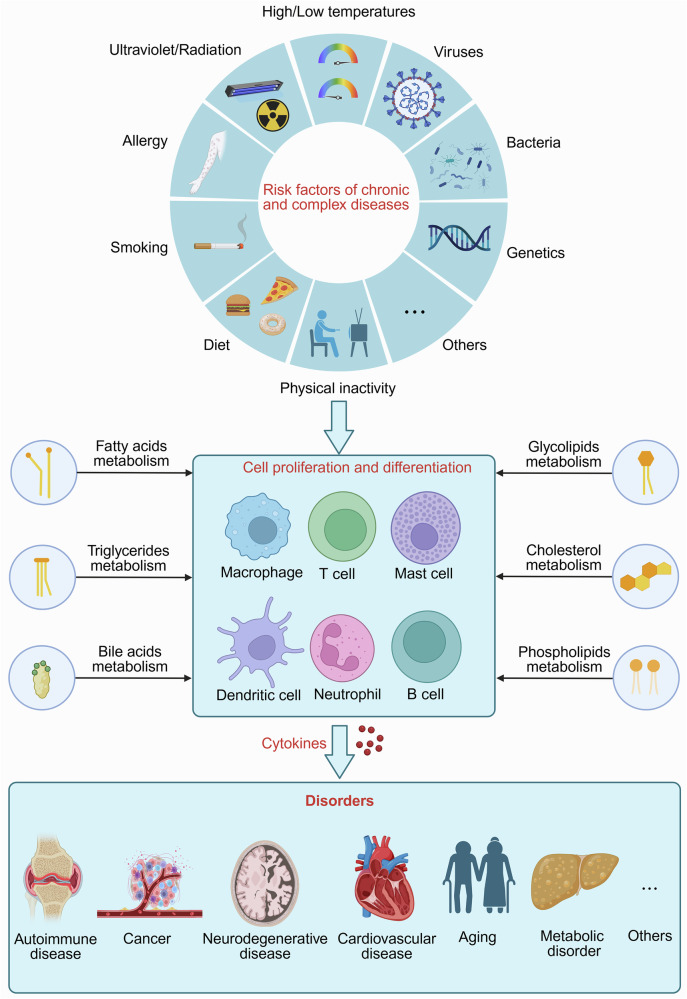
Etiological triggers of disease onset. Various factors, such as a high-fat diet, smoking, viral and bacterial infections, and genetic defects, can disrupt immune cell function and disturb immune homeostasis, resulting in various diseases, including cancer, aging, autoimmune diseases, NDDs, CVDs, etc. Lipid metabolism (e.g., FAs cholesterol, BAs phospholipids, glycolipids, and triglycerides) affects immune cell proliferation, differentiation, and the release of inflammatory mediators. Modulation of lipid metabolism may effectively mitigate the progression of these diseases. Created in BioRender

Lipids, including fatty acids (FAs), cholesterol, phospholipids, and triglycerides, are essential for maintaining physiological functions, such as energy storage, membrane structure, and cell signaling. Lipid metabolism, which involves the synthesis and degradation of lipids, plays a vital role in cellular homeostasis, and disruptions in lipid metabolism are associated with various pathological conditions.^1^ Recent research has highlighted lipids as key regulators of cellular function, particularly in immune cells, where lipid metabolism affects immune responses, cell phenotypes, metabolic pathways, and cytokine levels.^2–6^ This influence can profoundly affect their functions under both healthy and disease conditions (Fig. 1).

**Fig. 2:**
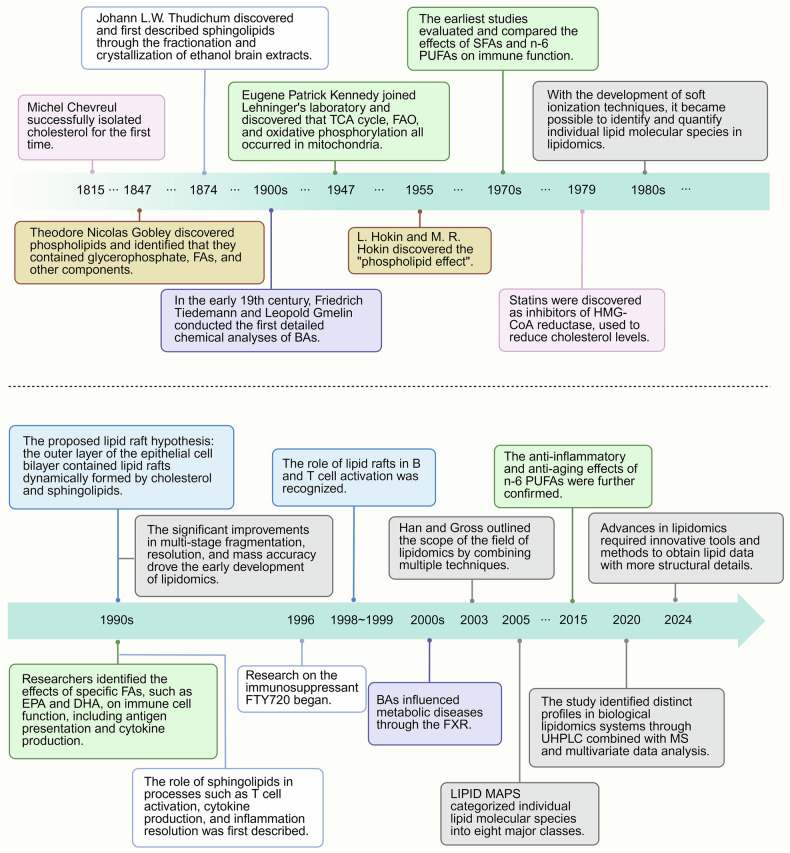
Timeline of lipid metabolism. The history of BAs is highlighted in pink boxes. New discoveries regarding phospholipids are shown in yellow boxes. The green boxes represent milestone events in the history of FAs. Key findings on lipid rafts are marked in blue boxes, whereas sphingolipid milestones are shown in white boxes. The gray boxes indicate historical developments in lipidomics. Over time, lipid metabolism has become a significant focus of research and interest. Created in BioRender

Historically, studies of lipid metabolism have evolved significantly over the years (Fig. 2). In the 19th century, important lipids such as bile acids (BAs), phospholipids, and sphingolipids were discovered,^7–10^ paving the way for further exploration of their biological roles. In the mid-20th century, advancements were made in studies of the role of cholesterol in metabolic diseases,^11^ fatty acid oxidation (FAO) pathways, and the “phospholipid effect”.^12^ The introduction of statins in 1979 revolutionized cardiovascular treatment,^13^ whereas breakthroughs in the 1990s elucidated the roles of sphingolipids in immunity and polyunsaturated FAs (PUFAs) in immune cell regulation.^14–16^ Advances in lipidomics, especially in mass spectrometry, have furthered the analysis of lipid structure and function, shedding light on the roles of lipids in inflammation and metabolism.^17–20^

Interestingly, lipid metabolism in immune cells is particularly dynamic, as it changes significantly during immune activation or inflammation. Unlike nonimmune cells, immune cells critically rely on lipid metabolism to support immune responses.^4,21–23^ For example, T cells and macrophages use FAO and oxidative phosphorylation in their resting state to maintain homeostasis, but during immune activation, they increase glucose metabolism and FAO to meet increased energy demands.^24–27^ In contrast, nonimmune cells, such as muscle cells, endothelial cells, and adipocytes, primarily utilize lipid metabolism for energy storage and basic functions, with less involvement in immune regulation.^28,29^

Clinically, targeting lipid metabolism in immune cells has shown therapeutic potential, particularly in inflammatory diseases, immune deficiencies, and cancer. For example, modulating FA metabolism in T cells can prevent exhaustion and increase the effectiveness of immune checkpoint inhibitors.^30,31^ Moreover, lipid metabolism in immune cells is closely linked to immune regulation in metabolic diseases, such as diabetes, obesity, and autoimmune diseases such as rheumatoid arthritis (RA).^32–34^ Targeting lipid metabolism in immune cells may help alleviate chronic low-grade inflammation, improve metabolic states, and enhance immune responses.^32–34^

This review aims to provide a comprehensive overview of how lipid metabolism influences immune cell behavior, particularly in macrophages, T cells, B cells, and neutrophils, under different physiological and pathological conditions. In this review, we first explore the roles of circulating lipids in regulating immune cell activity across various immunological contexts and disease environments. We then delineate the roles of membrane lipids in intracellular signaling and in shaping immune cell function. Additionally, we highlight the intricate interactions between intracellular signaling pathways and lipid metabolism under different conditions, as well as the underlying regulatory mechanisms involved. Finally, we present a thorough assessment of how lipid metabolism impacts various diseases, consolidating both preclinical and clinical evidence and emphasizing potential strategies targeting lipid pathways to modulate immune cell activity in disease management.

## Circulating lipids regulate immune cell responses

Circulating lipids, mainly FAs, cholesterol and cholesterol-derived metabolites, and BAs, are increasingly recognized as crucial sources of nutrients for cellular energy production and biomass generation.^35^ These lipids serve as essential reservoirs for energy and building blocks. Moreover, they function as signaling molecules crucial for both innate and adaptive immune responses. We summarized the roles of circulating lipids in regulating immune cell responses, especially in the response mediated by macrophages, T cells, B cells, neutrophils, and others (Table 1).

**Table 1:** Effects of lipid metabolites on immune cell activation and inhibition

Lipid sources or functions	Lipids/pathways	Cell types	Refs.
Lipids in microenvironment	SCFAs	M1 macrophage (-), M2 macrophage (+)	^50–53^
		M2 macrophage (-)	^53^
		Thymic Treg cell (+)	^46^
		Treg cell (+)	^117^
		Th1 and Th17 (+)	^49^
		Plasma B cell (+)	^44^
		IL-10^+^ B cell (+)	^45^
		Neutrophil (+)	^60,61^
		DC (+)	^62^
		Mast cell (-)	^63,64^
	LCFAs	Macrophage (+)	^83–86^
		CD4^+^ T cell (-)	^66–69^
		Treg (+), Th2 and Th1 (-)	^71,72,79^
		Th2 (+)	^521^
		CD8^+^ T cell (+)	^80^
		Th1 and Th17 (+)	^81^
		Neutrophil (+)	^75^
		DC (-)	^76^
		Mast cell (-)	^78^
	Cholesterol	Macrophage (+)	^88^
		Microglial (+)	^98,99^
		CD4^+^ and CD8^+^ T cells (+), Treg (-)	^100^
		CD4^+^ T cell and Tfh cell (+)	^101^
		Neutrophil (+)	^103^
		Neutrophil (-)	^105^
		DC (-)	^106^
		Eosinophil (+)	^107^
	Bile acids	M1 macrophage (+)	^109^
		Treg (+), Th17 (-)	^112–114,117^
		RORγ^+^ Treg (+)	^114,118^
		Neutrophil (-)	^120^
		Eosinophil (+)	^124^
		Mast cell (+)	^121^
		Mast cell (-)	^122,123^
Membrane lipids	Phosphatidic acids	Memory CD8^+^ T cell (+)	^135^
		Neutrophil (+)	^132,133^
		DC (+)	^147^
		Eosinophil (+), Basophil (+), Mast cell (+)	^149–151^
	S1P	M1 macrophage (-), M2 macrophage (+)	^158^
		CD4^+^ T cell (-)	^164,165^
		Neutrophil (+)	^167^
		Mast cell (+)	^168,169^
	Lipid rafts	B cell (+)	^173–175^
		T cell (+)	^185^
Intracellular programs	De novo lipid synthesis	CD8^+^ T cell (+)	^265^
		Treg (+), Th17 (-)	^268^
		Th2 (+)	^267^
		B cell (+)	^190,191^
		IL-10^+^ B cell (+)	^195^
		GC B cell (+)	^190^
		Neutrophil (+)	^207,208^
		DC (+)	^209^
	FAO pathway	Central memory CD8^+^ T cell (+)	^211–213^
		Effector CD8^+^ T cell (-)	^217^
		TRM T cell (+)	^218^
		GC B cell (+)	^222^
		Neutrophil (+)	^227,228^
		DC (+)	^229^
	Mevalonate pathway	B cell (+)	^192,202^
		IL-10^+^ B cell (+)	^195^
		Treg (-)	^233^
		Senescent T cell (+)	^31^
		Treg (+)	^204^
		Th1 (+), cytolytic T cell (+)	^206^
	Lipid droplets	Treg (-)	^241^
		Senescent T cell (+)	^233^
		DC (+)	^242,243^
SPMs	RvD1	IgM^+^ IgG^+^ B cell (+)	^475^
		IgE^+^ B cell (-)	^475^
		Treg (+)	^478^
		Th1, Th17 (-)	^477^
		Neutrophil (-)	^482^
	RvD2	Th1, Th17 (-)	^477^
		Treg (+)	^478^
	RvD3	Th1, Th17 (-)	^477^
	RvE1	Th17, Th2(-)	^479^
		Neutrophil (+)	^481^
	17-HDHA	IgM^+^ IgG^+^ B cell (+)	^475^
		IgE^+^ B cell (-)	^475^
	LXA4	Th1, Th17 (-)	^477^
	LXB4	Th1, Th17 (-)	^477^
	MaR1	Th1, Th17 (-)	^477^
		Treg (+)	^478^
	Protectins, Maresins, D-series resolvins	Macrophage (+)	^476^
Others	PPARs	VAT Treg (+)	^435^
		DC (-)	^257,458^
		Eosinophil (-)	^258^
	SREBPs	CD8^+^ T cell (+)	^265^
		Intratumoral Treg (+)	^203^
	FABPs	TRM T cell (+)	^219–221^
		Treg (+)	^222^

“+” represents activation, while “-” represents suppression

### Fatty acids

FAs are categorized according to their length into short-chain (SCFAs), medium-chain, long-chain (LCFAs), and very-long-chain FAs. They play integral roles in regulating immune cell function, inflammation, and overall immune system health.

#### SCFAs

**Fig. 3:**
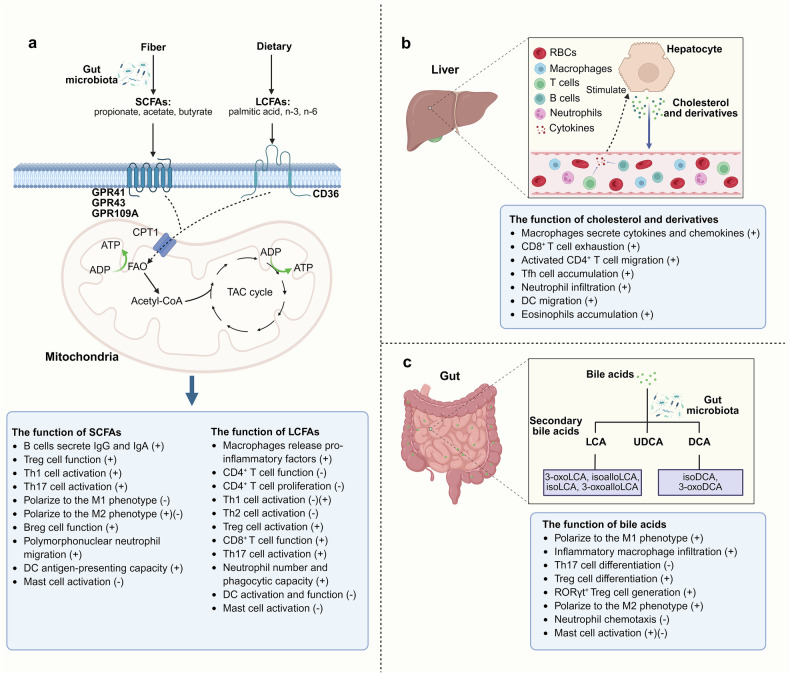
Regulation of immune cell functions by FAs, cholesterol, cholesterol derivatives, and BAs. **a** SCFAs originate from dietary fiber, whereas LCFAs are obtained primarily from the diet. SCFAs and LCFAs bind to the receptors GPR41, GPR43, GPR109A, and CD36, respectively, and enter the TCA cycle to regulate immune cell functions. **b** Cholesterol is predominantly synthesized in hepatocytes and released into the bloodstream, where it is absorbed by immune cells to elicit immune responses. **c** BAs, which originate from the liver, undergo metabolism by the gut microbiota to produce secondary BAs (e.g., LCA, DCA, UDCA), along with their derivatives (e.g., 3-oxoLCA/DCA, isoalloLCA, isoLCA/DCA, 3-oxoalloLCA). These metabolites play significant roles in mediating immune cell functions. The notation (+) indicates upregulation, whereas (-) indicates downregulation. Created in BioRender

SCFAs, such as acetate, propionate, and butyrate, are produced primarily by the gut microbiota through fermentation of dietary fiber (Fig. 3a).^36,37^ Dietary fiber provides an appropriate substrate for bacteria to produce SCFAs, which are directly associated with increased levels of SCFAs in the gut.^38^ In addition to promoting microbial diversity and SCFA production, a high-fiber diet can also decrease the levels of inflammatory markers, such as Janus kinase (JAK)/signal transducer and activator of transcription (STAT) and mitogen-activated protein kinase (MAPK) signaling pathways, in immune cells.^39^ In contrast, a low-fiber diet leads to decreased SCFA levels, impaired colonic mucus barrier function, and increased susceptibility of mice to pathogenic bacteria, such as *Citrobacter rodentium*.^40^ Therefore, Western diets are associated with lower fecal and serum SCFA levels and reduced microbial fermentation.^41^ Moreover, SCFAs have been shown to signal through surface-expressed free FA receptors or *via* G protein-coupled receptors (GPRs), such as GPR41, GPR43, and GPR109A, which are expressed on epithelial cells, adipose tissue, and immune cells.^42^ Both GPR41 and GPR43 bind acetate, propionate, and butyrate, whereas GPR109A is primarily activated by butyrate. The activation of GPR43 by SCFAs can have various effects depending on the cell type,^42^ which we will mention further below.

SCFAs can influence the recruitment of immune cells and affect the production of inflammatory mediators.^42,43^ For example, by inhibiting histone deacetylase (HDAC) activity, SCFAs promote mitochondrial oxidative phosphorylation as well as glycolysis, which are essential for robust plasma B cell generation.^44^ Moreover, SCFAs such as acetate enhance the function of IL-10-producing regulatory B (Breg) cells, contributing to immune regulation.^45^ The potential mechanism is that acetate induces Breg differentiation through its conversion into acetyl-CoA, which promotes the tricarboxylic acid (TCA) cycle and increases protein acetylation. This process undoubtedly promotes the production of IgG and IgA, thereby enhancing the humoral immune response. Moreover, SCFAs also increase the abundance of IgA-coated bacteria in the intestine, thereby regulating intestinal microorganisms, preventing dysbiosis, and maintaining intestinal immune homeostasis.^44^

The modulation of T cells by SCFAs is also crucial. For example, deregulation of T cell maturation is observed in preeclampsia, coincident with a reduction in maternal serum acetate levels. Similar phenomena have been noted in sterile mice, and such deficiencies can be rectified through supplementation with acetate,^46^ suggesting that SCFAs function in thymic T cell maturation, particularly regulatory T (Treg) cell maturation. Although there is ongoing debate regarding whether SCFAs can promote the differentiation of peripheral CD4^+^ T cells into forkhead box P3^+^ (Foxp3^+^) T cells, it is generally accepted that SCFAs can enhance the function of Treg cells either through GPR43 activation or by influencing histone acetylation levels.^47,48^ SCFAs are also recognized for their ability to increase the activities of T helper 1 (Th1) and Th17 cells, both of which contribute to preventing and combating infections, and for promoting immunity or immune tolerance according to the immunological context.^49^ Mechanistically, the effects of SCFAs on T cells do not rely on GPR41 or GPR43 but rather on the direct inhibitory activity of HDACs. The inhibition of HDACs by SCFAs in T cells increases the acetylation of p70 S6 kinase and the phosphorylation of rS6, thereby regulating the mammalian target of rapamycin (mTOR) pathway required for the generation of Th17 and Th1 cells.^49^

In macrophages, sodium butyrate significantly inhibits inflammation in lipopolysaccharide (LPS)-stimulated or other classically activated M1-polarized macrophages, inhibits M1 macrophage polarization and promotes oxidative phosphorylation to drive M2 macrophage polarization. This effect extends to regulating conditions such as muscle cell atrophy, nonalcoholic steatohepatitis (NASH), and macrophage-dependent intestinal immune homeostasis.^50–52^ Mechanistically, through HDAC inhibition, butyrate enhances the acetylation of the canonical nuclear factor kappa-light-chain enhancer of activated B cells (NF-κB) subunit p65 and its differential recruitment to pro-inflammatory gene promoters, independent of nuclear translocation, or through protein kinase B (Akt)/mTOR/forkhead box O3a (FoxO3a) and F-box protein 32/tripartite motif-containing protein 63 (FBOX32/TRIM63) pathways.^50,51^ Interestingly, while propionate and butyrate suppress M2 macrophage polarization and alleviate airway inflammation, acetate does not have the same effect and can instead abrogate M2 macrophage polarization in an asthma model and in human-derived macrophages.^53^ These findings suggest that SCFAs may exert a dual modulatory effect on macrophage activation. Moreover, exogenous acetate/propionate activates the cyclic GMP-AMP synthase-stimulator of interferon genes (STING)-type I interferon (IFN-I) axis through GPR43 signal transduction in macrophages. This activation protects against enteric virus infection in mice by increasing intracellular calcium (Ca^2+^) and mitochondrial antiviral signaling protein-dependent mitochondrial DNA release.^54^

Neutrophil extracellular traps (NETs) are mesh-like structures released by neutrophils under specific stimuli, such as inflammatory mediators, immune complexes, pathogenic microorganisms, and external environmental factors. NETs are composed primarily of DNA, histones, and various antimicrobial proteins, such as elastase and myeloperoxidase.^55,56^ The formation of NETs can be divided into two pathways: one is cell death-associated NET formation (also called NETosis), which involves cell membrane rupture and nuclear membrane disintegration and is usually accompanied by cell death; the other is the nonclassical pathway of NETosis, which involves chromatin extrusion and the release of granule proteins without cell death.^56^ NETs play a critical role in the initiation and perpetuation of systemic autoimmune diseases as well as in driving inflammatory responses that lead to organ damage.^57,58^ Studies have shown that SCFAs regulate the transepithelial migration of polymorphonuclear neutrophils and the formation of NETs at different concentrations. Acetate promotes polymorphonuclear neutrophil migration at high concentrations, whereas propionate and butyrate significantly induce both polymorphonuclear neutrophil migration and NETosis at specific concentrations.^59^ These results suggest that SCFAs enhance neutrophil function by stimulating their migration to inflamed areas, thereby increasing the activity of platelet-activating factor and exerting anti-inflammatory effects.^60,61^

With respect to other immune cells, SCFAs regulate immune balance and antitumor effects through HDAC inhibition, thereby enhancing the antigen-presenting capacity of dendritic cells (DCs).^62^ Moreover, SCFAs such as butyrate and pentanoate inhibit mast cell activation and reduce IgE-mediated degranulation by suppressing HDAC activity.^63,64^

Overall, SCFAs maintain immune homeostasis primarily by regulating cell differentiation, polarization, migration, and the immune response in various immune cells. They enhance immune responses by regulating T cell differentiation and function, promoting M2 macrophage polarization while inhibiting M1 macrophage polarization to exert anti-inflammatory effects, enhancing B cell differentiation and humoral immune responses, supporting neutrophil migration and function, promoting DC antigen presentation, and inhibiting mast cell activation.

#### LCFAs

Most LCFAs are present in most fats in our diet, such as corn oil, olive oil, and chicken fat, while a small amount is synthesized de novo (Fig. 3a). Upon consumption, LCFAs integrate into the cell membrane, influencing membrane fluidity and the immune response.^65^ LCFAs are made up of three types of PUFAs: unsaturated FA-, monounsaturated FA (MUFA), and saturated FA (SFA), according to their chemical structure.

PUFAs can modulate pathways in T and B cells, as observed in preclinical models. For example, fish oils are rich in n-3 long-chain PUFAs, such as docosahexaenoic acid (DHA) and eicosapentaenoic acid (EPA), both of which can influence T cell function by inhibiting signal transduction through the T cell receptor (TCR) and CD28, thus diminishing CD4^+^ T cell function.^66^ In addition, EPA, DHA, and arachidonic acid have been reported to directly become part of the cell membrane and decrease CD4^+^ T cell proliferation ex vivo and in vitro via the modulation of metabolism and inflammation.^67–69^ An in vitro study demonstrated that DHA can inhibit major histocompatibility complex class II (MHC-II) expression, the expression of costimulatory molecules (CD40, CD80, and CD86), and the production of inflammatory cytokines (IL-6 and IL-12p70), thereby suppressing CD4^+^ T cell activation.^70^ Furthermore, DHA and EPA can alleviate cow milk and peanut allergies in mice by reducing IgE, IgG1, and IgG2a levels, promoting Treg generation, and suppressing Th2 and Th1 cell activation.^71,72^ Notably, a soy diet rich in n-6 PUFAs increases Th1-like responses but decreases Th2-like responses.^73^ However, increasing the intake of n-3 long-chain PUFAs can mitigate this effect, highlighting the necessity of the n-3:n-6 PUFA ratio in dietary immune modulation.^71^

Furthermore, studies have shown that the levels of PUFAs in young and middle-aged individuals are significantly correlated with neutrophil function.^74^ Among these, n-3 PUFAs increase both the number and percentage of neutrophils during the perinatal period, thereby enhancing their ability to combat pathogens.^75^

In DCs, PUFAs such as conjugated linoleic acid, n-3, and n-6 FAs inhibit their activation and function.^76^ Specifically, n-6 PUFAs promote immune suppression by shifting DC metabolism toward glycolysis, thereby reducing immune stimulation.^77^ Furthermore, n-3 PUFAs inhibit Fc ε receptor I (FcεRI)-mediated mast cell activation, thereby reducing histamine levels.^78^

Additionally, MUFAs such as oleic acid improve human Treg functions by boosting FA oxidation-driven oxidative phosphorylation metabolism. This process forms a positive feedback loop that enhances Foxp3 expression and STAT5 phosphorylation.^79^ In addition, dietary trans-oleic acid, rather than its cis isomer oleic acid, can enhance basal or IFN-γ-stimulated MHC-I expression by upregulating MHC-I through nucleotide-binding oligomerization domain (NOD)-like receptor family caspase recruitment domain domain-containing 5 (NLRC5), promoting tumor antigen presentation and enhancing CD8^+^ T cell-mediated cytotoxicity.^80^

Moreover, SFAs such as lauric acid expand Th1 and Th17 cell populations by activating the p38/MAPK pathway,^81^ and contribute to a more severe course of experimental autoimmune encephalomyelitis (EAE), an animal model of multiple sclerosis (MS).^81,82^ In the context of macrophages, several studies have extensively documented the proinflammatory effects of palmitic acid. One study revealed that palmitic acid serves as a toll-like receptor (TLR) agonist, stimulating macrophages to release TNF-α, IL-1β, and IL-6 by enhancing TLR-induced signaling pathways.^83–87^

Overall, LCFAs expand Th1 and Th17 cell populations by activating the p38/MAPK pathway, thereby promoting immune responses. They stimulate macrophages through enhancing TLR-induced signaling pathways and enhance Treg cell function by promoting FAO metabolism to facilitate immune tolerance. Additionally, LCFAs improve neutrophil function and suppress the activation of DCs and mast cells, thus regulating immunity.

### Cholesterol

The abundance of cholesterol and its biosynthetic intermediates in the body has a significant impact on immune cell function in various disease contexts.^88,89^ In the periphery, the primary site of de novo cholesterol synthesis is hepatocytes (Fig. 3b). Cholesterol synthesized in the liver is released into the bloodstream, where it is absorbed by target cells, thereby influencing immune responses within the target tissue.^88^ During injury or infection, cytokines released into the bloodstream stimulate cholesterol synthesis in the liver.^90–92^ The liver then releases cholesterol into the bloodstream, facilitating its transport to immune cells in the periphery.

Notably, immune cells, particularly macrophages, can accumulate cellular cholesterol by absorbing modified low-density lipoprotein (LDL), thereby enhancing TLR signaling.^88^ Increased TLR activity leads to increased levels of cytokines and chemokines, exacerbating inflammation and potentially triggering NOD-like receptor family pyrin domain-containing 3 (NLRP3) inflammasome activation.^93–95^ Cholesterol accumulation and exposure to exogenous cholesterol can enhance the activation of inflammasomes in macrophages. Treatment of cultured macrophages with cholesterol crystals leads to rapid phagocytosis of the crystals, which are then stored in lipid droplets (LDs). These cholesterol-rich LDs subsequently drive the dose-dependent secretion of IL-1β, a process that relies on caspase-1 and NLRP3 activation.^96^ Studies have shown that this process is dependent on intracellular complement component (C) 5aR1 signaling, where C5a binds to receptors on the mitochondria, triggering ROS production and providing one of the necessary activation signals for inflammasome assembly and IL-1β secretion, especially in response to sterile inflammation induced by cholesterol crystal exposure.^97^ Furthermore, the absorption of cholesterol by neighboring neurons and microglia in neuroimmune macrophages influences the production of amyloid and neuroinflammatory cytokines.^98,99^

In the tumor microenvironment, cholesterol can cause endoplasmic reticulum stress, which promotes CD8^+^ T cell exhaustion and ultimately leads to uncontrolled tumor growth. Elevated plasma cholesterol levels disrupt T cell homeostasis, contributing to inflammation in patients with hypercholesterolemia.^100^ The oxysterol 7α,25-dihydroxycholesterol, a cholesterol derivative, signals through Epstein–Barr virus-induced gene 2 (EBI2, also known as GPR183) to facilitate the migration of activated CD4^+^ T cells to the interface between the B cell follicles and T cell zones in the spleen. This process enhances the accumulation of T follicular helper (Tfh) cells, thereby initiating humoral immunity.^101^

A high level of cholesterol can affect the normal function of neutrophils. Research has shown that a high-cholesterol diet induces neutrophil infiltration, which plays a key role in liver injury through myeloperoxidase activity,^102^ with 7-ketocholesterol being particularly important in this process. Similarly, cholesterol accumulation in bone marrow cells activates the NLRP3 inflammasome, enhancing neutrophil accumulation and the formation of NETs in atherosclerotic plaques.^103^ Promoting cholesterol efflux, such as through treatment with liver X receptor (LXR) agonists, can inhibit neutrophil recruitment in a sterile peritonitis mouse model.^104^ Interestingly, another cholesterol derivative, cholesterol sulfate, can directly act on inflammatory neutrophils, preventing excessive intestinal inflammation by inhibiting the Rac activator dedicator of cytokinesis 2 (DOCK2).^105^

For other immune cells, increased cholesterol levels inhibit DC migration to lymph nodes, while reducing cholesterol levels can partially reverse these migration defects.^106^ Studies have also shown that mice fed a high-cholesterol diet have a greater number of eosinophils in their bronchoalveolar lavage fluid, with elevated levels of IL-5, prostaglandin E2 (PGE_2_), and monocyte chemoattractant protein-1 (MCP-1), which enhances allergic inflammation in the lungs.^107^

Cholesterol promotes inflammation and IL-1β secretion in macrophages, triggers T cell exhaustion in CD8^+^ T cells, and enhances immune responses in B cells by facilitating T cell migration and humoral immunity. In addition, cholesterol affects the function of other immune cells through multiple pathways, including promoting immune cell infiltration, activating inflammatory pathways, and regulating cytokine secretion, which may exacerbate inflammatory responses. Overall, given their multiple functions in immune cells, sterols may become potential targets for the development of immunotherapy in the future.

### Bile acids

BAs are cholesterol metabolites that are abundantly stored in the mammalian intestine and promote lipid absorption in the intestine. BAs produced by the liver undergo metabolic transformation by the intestinal microbiota to produce enteric BAs such as lithocholic acid (LCA) and deoxycholic acid (DCA) (Fig. 3c). These derivatives play pivotal roles in immune cell biology.

As secondary BAs, DCA and LCA can enhance hematopoiesis in the bone marrow. Treatment of bone marrow cells with DCA and LCA preferentially expands immune phenotypes and functional colony-forming units, such as granulocyte‒macrophage progenitor cells.^108^ Among them, DCA enhances macrophage polarization toward the M1 phenotype, partly via TLR2 transactivation by the M2 muscarinic acetylcholine receptor, leading to increased levels of proinflammatory cytokines.^109^ In line with these findings, supplementation with DCA significantly promotes the infiltration of inflammatory macrophages and exacerbates the progression of colitis.^109,110^ However, variants of LCA, 3-oxoLCA and isoLCA have been identified as TGR5 agonists that promote M2 polarization of macrophages and have a positive effect on alleviating inflammation.^111^

Notably, BAs not only influence macrophage polarization but also play an important role in T-cell expansion. Derivatives of LCA and DCA act as crucial signaling molecules that modulate Th17 and Treg expansion, thereby reshaping gut inflammation.^112–114^ The variants of LCA, including iso-, 3-oxo-, allo-, 3-oxoallo-, and isoalloLCA, are formed through the collaborative action of 5α/β-reductase and 3α/β-HSDH.^115,116^ Among these variants, 3-oxoLCA directly interacts with retinoic acid receptor-related orphan nuclear receptor gamma t (RORγt), hindering Th17 cell differentiation. Conversely, isoalloLCA promotes the generation of Tregs by inducing mitochondrial ROS that increases Foxp3 expression.^112^ Unlike butyrate, which enhances Treg differentiation *via* the Foxp3 conserved noncoding sequence 1 (CNS1) enhancer, isoalloLCA-induced Treg differentiation is independent of the vitamin D receptor and farnesoid X receptor (FXR) CNS3.^112,117^ Another study revealed that isoalloLCA increases nuclear receptor 4 group A1 (NR4A1) binding at the Foxp3 locus, increasing Foxp3 transcription and promoting Treg differentiation.^113^ Therefore, an engineered consortium producing isoDCA that stimulates RORγt^+^ Tregs has been established in the gut *via* a CNS1-dependent mechanism.^114^ Another study indicated that disrupting the bile salt hydrolase of the Bacteroides genus can impair the intracellular dissociation of BAs, significantly reducing the induction of colonic RORγt^+^ Treg cells.^118^ Consistent with these findings, restoring the intestinal BA pool can increase the RORγt^+^ Treg population and improve the susceptibility of the host to inflammatory bowel disease (IBD) through BA nuclear receptors.^118^ Additionally, isoDCA can increase Foxp3 transcription by suppressing the immunostimulatory characteristics of DCs and, in turn, promote the proliferation of peripheral Tregs in the colon.^114^ Depletion of FXR in DCs mimics the transcriptional profile induced by isoDCA and augments peripheral Treg expansion, suggesting that the crosstalk between FXR and isoDCA is fundamental for maintaining the anti-inflammatory DC phenotype.^114^

In *H. pylori*-positive patients, a significant negative correlation has been observed between BA concentrations and the histological score of monocyte/neutrophil infiltration.^119^ Chenodeoxycholic acid (CDCA) has been previously shown to inhibit neutrophil chemotaxis and Ca^2+^ influx by competing with N-formyl-Met-Leu-Phe for binding to formyl peptide receptor 1 (FPR1). Similarly, DCA has been shown to suppress neutrophil chemotaxis and Ca^2+^ mobilization, and it is also believed to inhibit FPR1 signaling.^120^

Hydrophilic and lipophilic BAs exert distinct effects on immune cells. In mast cells, lipophilic dihydroxy BAs (such as CDCA and DCA as well as their glycine and taurine conjugates) directly activate mast cells, leading to histamine release.^121^ In contrast, hydrophilic BAs (such as ursodeoxycholic acid (UDCA) and ursocholic acid and their conjugates) suppress mast cell activation, thereby alleviating bile duct-related damage, fibrosis, and inflammation in cholestatic diseases.^122,123^ However, unlike mast cells, taurine-conjugated CDCA and taurine-conjugated UDCA can activate human eosinophils at specific concentrations.^124^

Overall, although BAs are involved in immune regulation in various immune cells, their mechanisms and effects differ significantly: in macrophages, BAs affect inflammation by modulating polarization states, whereas in T cells, BAs regulate immune tolerance and response by modulating cytokine production and differentiation. For other immune cells, hydrophilic BAs alleviate inflammation by inhibiting the activation of neutrophils, DCs, and mast cells, whereas lipophilic BAs modulate immune responses by activating mast cells.

## Membrane lipids regulate immune cell responses

It is well known that phospholipids, sphingolipids, and cholesterol, as well as the functional membrane microdomains enriched with specific lipids, such as lipid rafts, are primarily components of cell membranes. These membrane lipid structures, functions, and signaling pathways are intricately connected to the expansion and activation of immune cells.

### Phospholipids

**Fig. 4:**
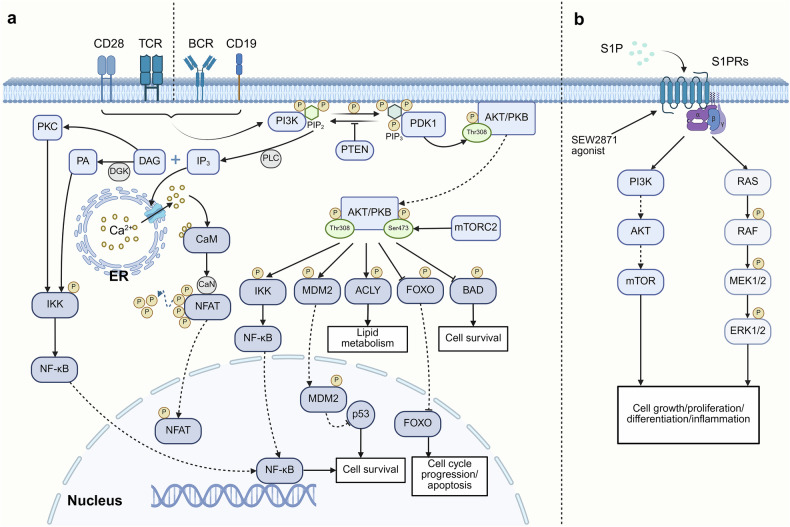
Phospholipids mediate signaling in immune cells. **a** Antigens, costimulatory signals, cytokines, and other factors activate PI3K, which phosphorylates the metabolite PIP2 to phosphatidylinositol-3,4,5-trisphosphate (PIP3). Conversely, PTEN acts in the opposite direction, terminating the PI3K signaling pathway. PIP3 recruits phosphoinositide-dependent kinase-1 (PDK1), activating AKT via phosphorylation at Thr308. In addition, mTORC2 phosphorylates AKT at Ser473. Activated AKT dissociates from the cell membrane to phosphorylate target proteins, such as BAD, FOXO, ACLY, MDM2, and IKK, influencing lipid metabolism, survival, apoptosis, differentiation, and other cellular responses in immune cells. PIP2 activates PLC, producing DAG and IP3. IP3 releases Ca^2+^ ions from the endoplasmic reticulum, leading to the progressive activation of CaM and CaN. CaN hydrolyzes and dephosphorylates NFAT, ultimately promoting its entry into the nucleus to mediate immune cell proliferation and differentiation. DGK converts DAG to PA and, together with PKC, initiates downstream signaling cascades that promote NF-κB entry into the nucleus, modulating the immune response. **b** Externally transported S1P binds to S1PRs via autocrine or paracrine signaling, regulating pathways such as the PI3K/AKT and MAPK pathways. Created in BioRender

Phospholipids constitute a diverse group of lipids that regulate intracellular signaling (Fig. 4a). Upon activation, TCRs and B cell receptors (BCRs) initiate a signaling cascade that involves the activation of phospholipase C (PLC). This enzyme hydrolyzes phosphatidylinositol 4,5-bisphosphate (PIP2) into inositol triphosphate (IP3) and diacylglycerol (DAG). IP3 then promotes sustained Ca^2+^ influx, leading to an increase in the intracellular Ca^2+^ concentration. This process facilitates the translocation of activated T cell nuclear factor (NFAT) into the nucleus, leading to NFAT-mediated gene transcription initiation.^125^ Notably, NFAT family members (including NFATc1, NFATc2, and NFATc4) regulate T cell proliferation and differentiation.^126^ NFAT1 promotes Th1 differentiation and IFN-γ secretion, influencing the Th1/Th2 balance.^127,128^ Mice lacking NFAT1 and NFAT4 exhibit increased Th2 cell differentiation and cytokine levels.^126,129^ Additionally, NFAT2 deficiency reduces IL-4 secretion and impairs Th2 cell differentiation.^130,131^ These studies highlight the crucial role of NFAT in T cell differentiation.

NFAT signaling plays crucial roles in the activation, function, and migration of neutrophils. It is activated in neutrophils by various stimuli, including the binding of dectin-1 with fungal ligands such as yeast glucan. This activation triggers the expression of inflammatory genes, including *Il10* and *Cyclooxygenase 2* (*Cox2*), enhancing neutrophil responses.^132^ Inhibition of calcineurin (CaN)-NFAT signaling *via* drugs such as Ca^2+^/calmodulin (CaM)-dependent phosphatase inhibitors has been shown to impair neutrophil function, thereby increasing susceptibility to bacterial and fungal infections. These effects are attributed to the disruption of NFAT signaling in myeloid cells (including neutrophils), which in turn impairs pathogen clearance and immune responses.^132,133^

The diacylglycerol kinase (DGK)-mediated conversion of DAG to phosphatidic acid (PA) activates NF-κB, which is crucial for T cell function. DGK overexpression leads to defects in TCR signaling, while its deficiency promotes T cell expansion and IL-2 secretion.^134^ Furthermore, DGK defects promote CD8^+^ T cell activation and cytokine production during viral clearance but inhibit memory CD8^+^ T cell proliferation upon reinfection.^135^ These findings illustrate the duality of DGK in effector and memory CD8^+^ T cells.

Phosphoinositides are glycerophospholipids, with phosphoinositide 3-kinase (PI3K) serving as a crucial lipid kinase. By facilitating the PI3K/AKT signaling pathway, these lipids selectively recruit signaling proteins to the cell membrane to modulate the development and function of T and B cells.^136,137^ In B cells, once phosphorylated, AKT regulates protein expression, including B cell lymphoma 2-associated agonist of cell death (BAD), FOXO, mouse double minute 2 homolog (MDM2), IκB kinase (IKK), and ATP citrate lyase (ACLY), which control cell cycle progression, survival, metabolism, differentiation, lipid synthesis, and other functions.^137,138^ Notably, as a transcription factor crucial in various physiological and pathological processes, the transcriptional activity of FOXO can be inhibited when it is phosphorylated by AKT,^139^ eventually promoting B-cell proliferation and survival.^140,141^ In line with these results, FOXO deletion affects key B-cell genes, including *early B-cell factor (EBF1)*, the *IL-7 receptor, recombination-activating genes (RAG1 and 2), activation-induced cytidine deaminase (AID), L-selectin*, and *B-cell linker protein (BLNK)*, leading to B cell development.^142^ By deleting AKT1/2 or PI3KR1, FOXO expression can be disrupted, resulting in the development and maturation of B cells, particularly marginal zone B cells.^143–145^ For other immune cells, mice lacking FOXO 3a produce increased Th1- and Th2-secreted cytokines.^146^ Moreover, the inhibition of PI3K-AKT phosphorylation, which enhances FOXO1 expression, can suppress DC maturation.^147^

PI3K/AKT also has crucial functions in other immune cells. The inhibition of the PI3K/AKT signaling pathway regulates hypoxia-inducible factor-1α, affecting the expression of lactate dehydrogenase A, thereby inhibiting glycolysis in neutrophils, reducing their chemotaxis and phagocytic functions, and leading to immune suppression.^148^ Similarly, downregulation of the PI3K/AKT pathway inhibits the proliferation, migration, and degranulation of eosinophils, basophils, and mast cells in mice.^48,149–151^ These findings underscore the value of PI3K/AKT pathway signaling proteins as targets for the treatment of immune cell-mediated autoimmunity and malignancy.

In T cells, phospholipids activate signaling and gene transcription to regulate immune responses following TCR activation, particularly through the activation of the IP3 and NFAT transcription factors, which control T cell proliferation and differentiation. In B cells, phospholipids regulate immune tolerance by modulating cell survival, metabolism, and differentiation through the PI3K/AKT signaling pathway and the FOXO transcription factor, which governs metabolic and cell growth signals. In neutrophils, phospholipids regulate immune responses by activating the NFAT and PI3K/AKT signaling pathways, which control glycolysis and cell migration. Additionally, this pathway plays an important role in the maturation and activation of DCs and regulates the proliferation, migration, and degranulation of eosinophils, basophils, and mast cells.

### Sphingolipids

Sphingolipids, although constituting a relatively minor portion (approximately 5%) of membrane lipids, play indispensable roles. As a sphingolipid metabolite, sphingosine-1-phosphate (S1P) can activate a series of downstream signaling molecules and is a bioactive substance that regulates cell physiological function and the immune response (Fig. 4b).^152^ The biological effects of S1P are mediated primarily through interactions with members of the GPR family, namely, S1P receptors (S1PRs) 1–5, to regulate the differentiation of immune cells and the secretion of proinflammatory cytokines and eicosanoids.^153,154^ The S1P-S1PR pathway governs the movement and homing of various immune cells through signaling mechanisms such as the PI3K/AKT and MAPK/extracellular signal-regulated kinase (ERK) pathways.^155,156^

Previous studies have shown that S1PR1 and S1PR2 are expressed in different populations of macrophages and monocytes.^157^ In peritoneal macrophages from LDL receptor (LDLR)-deficient mice, the activation of S1PRs significantly reduces the levels of inflammatory TNF-α, TNF-R, and IL-6 upon LPS stimulation.^158^ In addition, S1PR activation can change the activation state and function of macrophages, changing them from the M1 type to the M2 type, controlling inflammation and thus alleviating atherosclerotic lesions.^158^ Similarly, a specific agonist of S1P or S1PR1, SEW2871, significantly inhibits markers of LPS-induced M1-type responses, such as TNF, C-C motif chemokine ligand 2 (CCL2), and IL-12, further demonstrating the beneficial effects of S1PR activation on the body.^157,159^

Interestingly, another receptor, S1PR2, which is found in peritoneal macrophages in mice, is more effective at inhibiting the LPS-induced inflammatory response. Compared with macrophages from wild-type mice, S1PR2-deficient mice eliminate the effects of S1P and SEW2871 on macrophages,^157,159^ indicating that S1PR2 contributes to the functions of S1P and SEW2871. On the other hand, acting as a survival messenger, S1P is secreted by sphingosine kinase 1 (Sphk1) upon stimulation by apoptotic cells and confers a protective effect on macrophages, thereby preventing their early apoptosis.^160^

S1P also significantly impacts the immune responses mediated by T and B cells. The S1P-S1PR1 pathway promotes CD4^+^ T cell differentiation into Th1/Th17 cells but has a negligible effect on the cytotoxic T lymphocyte activity of allogeneic CD8^+^ T cells.^161^ Th17 cells are excreted from the intestine in an S1PR1-dependent manner and subsequently migrate to the kidney *via* the CCL20/C-C motif chemokine receptor 6 (CCR6) axis, thereby causing nephritis.^162^ Furthermore, S1PR inhibits extrathymic and innate Treg cell production while driving Th1 development in a reciprocal manner, which is reciprocally regulated by S1P1-mTOR and the opposing TGF-β-Smad3 signaling.^163^ Targeted S1P therapy helps maintain the survival of both T and B cells, inhibits homeostatic proliferation, and suppresses cytokine production induced by TCR activation in CD4^+^ T cells.^164,165^

According to previous reports, S1P strongly promotes the migration and cytoskeletal remodeling of neutrophils in the bone marrow *via* S1PR1 or S1PR2.^166^ Blocking S1PR2 significantly reduces neutrophil infiltration in liver injury induced by bile duct ligation in mice. Thus, the S1P/S1PR system plays a crucial role in neutrophil recruitment. Additionally, S1P stimulates NETosis through receptors on neutrophils, and inhibiting S1P signaling can effectively prevent NETosis.^167^

In allergic diseases, changes in S1P levels can influence the differentiation and reactivity of mast cells. Mast cell activation requires Sphk activation and the secretion of S1P.^168^ Studies have shown that *Sphk2* knockout mice exhibit impaired mast cell degranulation,^169^ further highlighting the critical role of S1P in mast cell activation. Overall, S1P regulates polarization in macrophages, promotes immune responses in T cells, maintains immune homeostasis in B cells, and regulates migration and activation in neutrophils and mast cells.

### Lipid rafts

Plasma membrane lipid rafts are microdomains enriched in cholesterol and sphingolipids, constituting crucial components of the cell membrane. These cholesterol- and sphingolipid-rich regions form tightly packed, low-fluidity microdomains within the membrane, which participate in essential cellular processes, including regulating immune cell activation by reorganizing receptor localization and facilitating signal cascades.^170^

**Fig. 5:**
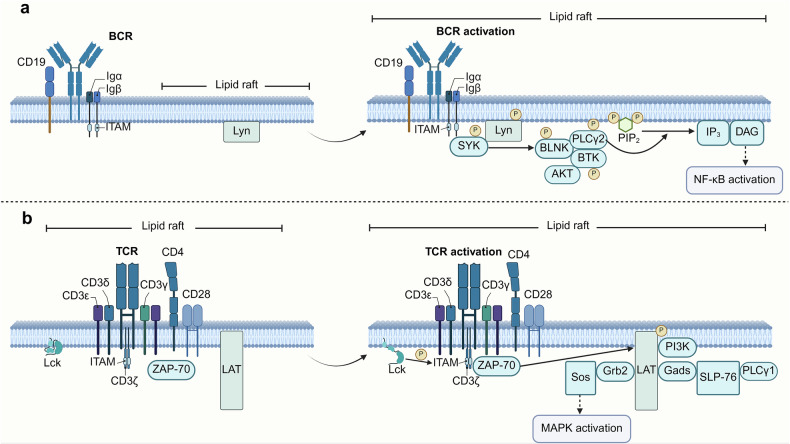
Activation of cell receptors and proximal signal transduction. **a** BCRs on the cell surface recognize and bind various forms of antigens, initiating an activation signal in B cells. This activation triggers a signaling cascade that results in complete B cell activation. CD19 serves as a critical coreceptor in this process, amplifying BCR signaling and enhancing downstream pathways such as the PI3K/AKT pathway, which promotes B cell proliferation and survival. **b** TCR activation relies on antigen-specific recognition and is further strengthened by auxiliary signals from CD3, coreceptors (such as CD4), and costimulatory signals from CD28, ultimately leading to T cell activation. Created in BioRender

In B cells, under resting conditions, BCR and CD19 exhibit low affinity for lipid rafts and are therefore located primarily in nonraft domains of the plasma membrane.^171^ Upon antigen binding, mature B cells’ BCRs are more effectively recruited into lipid rafts, initiating the primary signaling events required for B cell activation (Fig. 5a).^172^ The Src family kinase Lyn subsequently acts downstream of the BCR to trigger a signaling cascade, leading to full B cell activation.^173^ Upon BCR activation, the immunoreceptor tyrosine-based activation motifs (ITAMs) of Igα/Igβ undergo phosphorylation. This is followed by the phosphorylation of spleen tyrosine kinase (SYK) and BLNK, further propagating downstream signaling.^174^ Phosphorylated BLNK serves as a docking site for Bruton’s tyrosine kinase (BTK) and PLC-γ2, activating the NF-κB or PI3K/AKT signaling pathways, which promote B cell proliferation, differentiation, and antibody production.^174,175^ In immature B cells, even when antigens bind to the BCR, the complex fails to stably cluster within lipid rafts, resulting in inefficient signal initiation and often leading to failed activation or apoptosis.^172^ Additionally, studies have shown that Raftlin, a novel lipid raft linker protein, is critical for BCR-mediated signaling. The absence of Raftlin significantly reduces the levels of Lyn and ganglioside GM1 within lipid rafts, impairing tyrosine phosphorylation and Ca^2+^ signaling and thereby suppressing B cell activation.^176^

In T cells, TCRs are weakly associated with lipid rafts under resting conditions, but this association is significantly enhanced upon antigen stimulation.^177^ During T cell activation, the TCR complex aggregates with signaling molecules in lipid rafts, such as the Src family lymphocyte-specific protein tyrosine kinase (Lck), thereby increasing signal transduction (Fig. 5b). Upon activation, Lck phosphorylates the ITAM regions on the CD3 and ζ chains of the TCR complex.^178^ Phosphorylated ITAMs provide binding sites for the downstream signaling protein ZAP-70, which subsequently phosphorylates linkers for the activation of T cells (LATs).^179,180^ Phosphorylated LAT recruits and activates multiple downstream proteins, including GRB2-related adapter protein downstream of Shc (Gads), SH2 domain-containing leukocyte protein of 76 kDa (SLP-76), and PLC-γ1, further propagating the signaling cascade. Gads bind to phosphorylated LAT and SLP-76, forming the LAT-Gads-SLP-76 complex.^181^ Through this interaction, Gads facilitates the recruitment of SLP-76 into the LAT complex, enabling PLC-γ1 to bind and be further activated by SLP-76.^182^ This process is essential for the generation of Ca^2+^ and protein kinase C (PKC) signals, which drive T cell activation and functional responses. Additionally, growth factor receptor-bound protein 2 (Grb2) binds to the son of sevenless (Sos), localizing Sos near the membrane to activate the MAPK pathway.^183^ The integrity of lipid rafts is crucial for T cell activation. Reducing glycosphingolipid levels in CD4^+^ T cell lipid rafts impairs TCR signaling, thereby diminishing Th17 differentiation.^184^ Moreover, gangliosides, key components of lipid rafts, play distinct roles in the activation of CD4^+^ and CD8^+^ T cells.^185^ The loss of α2,6-sialylation disrupts TCR translocation to lipid rafts, suppressing CD4^+^ T cell activation and NF-κB expression, which leads to reduced production of proinflammatory cytokines and alleviates ulcerative colitis progression.^186^ Interestingly, cholesterol-rich lipid rafts on activated T cells may enhance viral entry and syncytium formation.^187^

In conclusion, lipid rafts play crucial roles in regulating immune cell signaling, activation, differentiation, migration, and functional execution in immune cells. Their integrity and functional abnormalities are closely associated with various diseases. Targeting specific components of lipid rafts may provide new strategies for immune regulation and disease treatment. However, compared with other lipid mediators, there has been relatively limited research on the role of lipid rafts in immune cell-based disease therapies. Future research should focus more on the key functions of lipid rafts.

## Intracellular reprogramming of lipid metabolism regulates immune cell responses

**Fig. 6:**
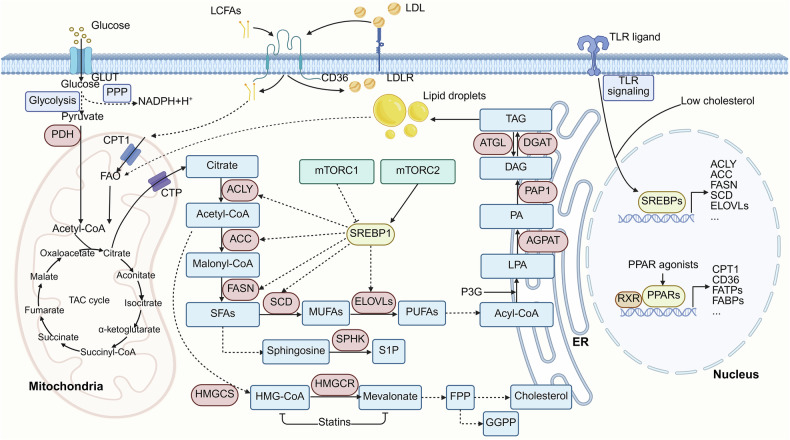
Intracellular lipid metabolism in immune cells. Intracellular lipid homeostasis is intricately regulated by multiple pathways, including glycolysis, the mevalonate pathway, the PPP, mTOR, and TLR signaling. Glucose is catabolized into acetyl-CoA and NADPH *via* glycolysis and the PPP, respectively. Acetyl-CoA enters the TCA cycle for FA synthesis (involving the enzymes ACLY, ACC, FASN, SCD, and ELOVLs), ceramide synthesis, and cholesterol production *via* the mevalonate pathway. NADPH is essential for lipid synthesis and energy supply. Both mTORC1 and mTORC2 activate SREBP1 expression and protein hydrolysis, with SREBP1 acting as an important transcriptional regulator affecting the activity of FA synthases (e.g., ACLY, ACC, and FASN). Excess FAs are converted into triglycerides through 1-acylglycerol-3-phosphate acyltransferase (AGPAT), phosphatidic acid phosphatase 1 (PAP1) and DGAT. Triglycerides accumulate and gradually form droplet lipids, which serve as storage sites for lipids. Lipid catabolism occurs *via* FAO in mitochondria, with FAs transported by CD36, and droplet lipids enter the TAC cycle to produce energy *via* FAO. FA, cholesterol, and TLR signaling mediate the expression of nuclear receptor SREBPs and PPARs, thereby modulating lipid synthesis, catabolism, and storage in immune cells. Created in BioRender

Intracellular lipid homeostasis relies on a precise balance of lipid synthesis, catabolism, and storage. The immune system defends the body by eliminating pathogens, which necessitates the rapid proliferation and differentiation of immune cells. This process requires increased lipogenic activity to provide essential building blocks and energy sources.^188,189^ Hence, any disruption in normal intracellular lipid synthesis and catabolism can significantly impair immune cell function. In this section, we elaborate on the critical role of intracellular lipid metabolism in directing immune cell differentiation and function (Fig. 6).

### Lipid synthesis

Recent research has emphasized that lipogenesis, which serves as a fundamental building block and energy source, is crucial for immune cell functionality and inflammatory responses.

Compared with resting B cells, B cells exhibit elevated expression of key FA biosynthetic genes, suggesting enhanced de novo synthesis. This includes the upregulation of genes encoding *acetyl-CoA carboxylase alpha (ACACA), elongase of very long chain FAs 1 (Elovl1), Elovl5, Elovl6, fatty acid synthase (FASN)*, and *stearoyl-CoA desaturase 2 (SCD2)*.^190^ When receiving activation signals, B cells redirect glucose to the pentose phosphate pathway (PPP) to produce nicotinamide adenine dinucleotide phosphate (NADPH) and utilize acetyl-CoA from glucose oxidation. ACLY catalyzes the conversion of citrate produced in the mitochondria into acetyl-CoA, which is then utilized in the cytoplasm for the synthesis of FAs and cholesterol. Elevated ACLY levels and activity are observed in B cells stimulated with LPS. Accordingly, inhibiting ACLY function in these cells suppresses proliferation, impairs endomembrane expansion, and decreases CD138 expression and B lymphocyte-induced maturation protein 1 (Blimp-1).^191^ Furthermore, SCD is crucial for converting SFAs into MUFAs. Additionally, intrinsic SCD, particularly oleic acid, is essential for early B-cell development and germinal center (GC) formation during immunization and influenza infection in vivo.^190^

Transcriptomic analysis revealed marked upregulation of genes linked to the mevalonate pathway following CD40-induced B cell activation.^192^ A mechanistic study revealed that the mevalonate pathway is essential for cholesterol synthesis in B cells, with its key enzyme, HMG-CoA reductase, being susceptible to targeted inhibition by statins.^193,194^ Geranylgeranyl pyrophosphate (GGPP), a significant intermediary in cholesterol biosynthesis, counteracts the inhibitory effects of statins on B cell antigen presentation.^192^ Moreover, Bregs rely on GGPP synthesis to facilitate IL-10 production triggered by TLR9 ligation. This process involves a GGPP-initiated phosphorylation cascade that modulates the PI3Kδ-AKT-glycogen synthase kinase-3 (GSK-3) pathway, enhancing Blimp-1-dependent IL-10 synthesis.^195^

For T cells, the response of CD8^+^ T cells to infection relies on the extraction of acetyl-CoA from citrate *via* ACLY.^196^ Upon IL-12 stimulation, CD8^+^ T cells maintain high levels of IFN-γ through an ACLY-dependent pathway.^197^ However, under nutrient restriction conditions, the inhibition of ACLY significantly impairs IFN-γ production and reduces CD8^+^ T cell viability.^198^ Modulating the expression of ACLY in T cells has emerged as a new strategy for treating diseases, especially intestinal inflammation.^199^ Moreover, CD8^+^ T cells show increased lipid storage due to elevated acetyl-CoA carboxylase (ACC) activity. Inhibition of ACC activity can modulate T cell metabolism, increase T cell survival and multifunctionality, and effectively suppress tumor growth.^200^ FASN, a key enzyme in FA synthesis, deficiency leads to elevated MHC-I levels and promotes the killing of cancer cells by tumor-infiltrating CD8^+^ T cells.^201^ Inhibition of FASN also results in a decrease in the survival rate of memory CD8^+^ T cells but has no significant effect on the survival of effector CD8^+^ T cells.^202^ Additionally, FASN plays an important role in the functional maturation of Treg cells; loss of FASN impairs Treg cell function and further suppresses tumor growth.^203^

Activation of the mevalonate pathway is also critical for maintaining T cell function and stability by inducing T cell proliferation and suppressing IFN-γ and IL-17A levels.^204^ In addition, GGPP enhances STAT5 phosphorylation to maintain the function of Treg cells through IL-2.^204^ However, the application of statin drugs inhibits the mevalonate pathway and suppresses the activity of Treg cells.^205^ Interestingly, inhibiting the mevalonate pathway can also induce Th1 and cytolytic T cell responses, enhancing the antitumor immune effect.^206^

FASN is also essential for maintaining mature neutrophils, and its deficiency is associated with increased neutrophil apoptosis.^207^ Additionally, in autophagy-related 7 (Atg7)-deficient myeloblasts, treatment with pyruvate alone or exogenous free FAs (such as linolenic acid or a mixture of unsaturated and SFAs) is sufficient to restore normal glucose metabolism and rescue the defective neutrophil differentiation process.^208^

For DC maturation and function, DCs synthesize FAs from nonlipid precursors such as glucose and glutamine, which are converted into citrate and then into acetyl-CoA. Acetyl-CoA is further processed by ACC1 into malonyl-CoA, which is elongated by FASN to form palmitic acid. The transcription factor sterol regulatory element-binding protein 1 (SREBP1) regulates genes involved in this process. FASN supports the remodeling of the endoplasmic reticulum and Golgi apparatus, which is crucial for DC maturation. Inhibition of FASN *via* ACC1 or FASN inhibitors impairs DC maturation and antigen-presenting functions. Additionally, FASN plays a role in DC-mediated antitumor immunity, and inhibiting FASN can restore DC function in the tumor microenvironment.^209^ In summary, targeting lipid synthesis to regulate immune cells has potential for treating the progression of various diseases.

### Lipid catabolism

FAO is a critical catabolic pathway responsible for breaking down FAs into acetyl-CoA, a substrate for the mitochondrial TCA. Carnitine palmitoyl transferase 1 (CPT1) plays a crucial role in FAO by promoting the transport of cytoplasmic FAs into mitochondria. Modulating FAO presents a promising strategy for addressing diseases associated with dysregulated fat and cholesterol metabolism, particularly in macrophages, as highlighted in several studies.^210,211^ Previous studies have implicated the blockade of CPT1 in the progression of atherosclerosis in macrophages due to increased expression of CD36, a scavenger receptor involved in LDL uptake and subsequent lipid accumulation.^210^

In T cells, the activation of FAO is correlated with increased AMPK activity, which supports the development of central memory CD8^+^ T cells, a subset that circulates through secondary lymphoid organs.^212,213^ Unlike effector CD8^+^ T cells, central memory CD8^+^ T cells primarily utilize de novo synthesized triacylglycerides from glucose for FAO, which rely on lysosomal acid lipase.^202^ Unlike IL-15 signaling, which enhances CPT1a expression to promote FAO, IL-7 signaling stimulates glycerol uptake for triacylglycerol (TAG) synthesis and FAO, contributing to central memory CD8^+^ T cell longevity.^214,215^ While genetic deletion of *CPT1a* in T cells does not impair memory CD8^+^ T cell formation, treatment with etomoxir, a CPT1a inhibitor, significantly reduces mitochondrial oxidative function. This contrast suggests that CPT1a-independent pathways may compensate for memory T cell generation, whereas etomoxir’s broader inhibition of FAO disrupts mitochondrial integrity, which is essential for this process.^213,214,216^

In obesity-related breast tumor models, FAO can be increased upon STAT3 activation, which reduces the effector capabilities of CD8^+^ T cells.^217^ However, despite exhibiting dysfunction, CD8^+^ T cells from an obesity-related mouse colon carcinoma 38 tumor model did not show elevated *FAO* genes, suggesting a context-dependent influence of the tumor environment on lipid metabolism regulation in tumor-infiltrating T cells.^218^ Thus, FAO is linked to the formation and function of memory T cells under inflammatory conditions.

Compared with other memory T cell subsets, TRM cells exhibit greater extracellular lipid uptake and depend on FAO for their generation and survival.^219^ Fatty acid-binding proteins (FABPs) 4 and 5 regulate lipid transport and trafficking within cells. The absence of these proteins reduces mitochondrial oxygen consumption and diminishes virus-specific TRM cell accumulation in the skin, whereas FABP1 supports TRM cell accumulation in the liver.^219,220^ This metabolic shift toward FAO promotes TRM cell longevity, which is associated with improved antitumor immunity in gastric adenocarcinoma.^221^ Furthermore, FABP5 in Treg cells restrains mitochondrial DNA-triggered type I IFN signaling and promotes IL-10 production, highlighting the role of FABPs in modulating T cell responses within tissue microenvironments, including tumors.^222^

While rapidly dividing cells, such as activated T lymphocytes, predominantly rely on aerobic glycolysis for energy generation,^223,224^ the specific energy derivation mechanism in GC B cells remained unclear until recent studies. A groundbreaking investigation revealed that GC B cells primarily utilize FAO instead of glycolysis for energy production. Using isotope tracing techniques, researchers observed increased FA uptake by GC B cells in vivo and demonstrated that GC B cells cultured in vitro preferentially metabolize FAs over glucose to generate significant amounts of acetyl-CoA.^225^

Interestingly, FAO inhibition preferentially disrupts the recruitment of immature neutrophil populations (Ly6Glo/dim) that are influenced by pathogens,^226^ whereas neutrophil trafficking to infection sites requires CPT1a-dependent FAO.^227^ In neutrophils, FAO is particularly important during their differentiation, where autophagy plays a crucial role by degrading LDs to provide free FAs, thus maintaining metabolic energy balance. This autophagy-regulated FAO-oxidative phosphorylation pathway appears to be essential for providing ATP to meet the energy demands of the differentiation process.^228^

FAO also plays a critical role in DC function, particularly in the development of DC subsets and the regulation of immune tolerance. By breaking down FAs, FAO generates metabolic products such as acetyl-CoA, which provide energy for DCs and support their differentiation and activation.^229^ FAO not only influences the differentiation of DCs but also regulates their function. ROS derived from FAO may impair DC antigen presentation, but antioxidants can mitigate this negative effect.^230^ The regulatory mechanisms of FAO and its effects on different DC subsets require further investigation to better understand its complex role in immune responses. These findings indicate that FAO mediates the growth, development, activation and function of immune cells.

### Lipid storage

Within immune cells, LDs serve as structural indicators of the immune response.^231,232^ Treg cells exhibit a higher LD content than conventional T cells do.^233^ Diacylglycerol acyl transferase (DGAT) catalyzes the reaction between DAG and FAs, mediating the formation of LDs. Inhibiting DGAT1 activity disrupts Foxp3 induction, indicating the potential role of LDs in the formation or maintenance of Treg cells.^233^ Moreover, the function of T cells can be indirectly influenced by the LD content of other immune cells. For example, enhancing the synthesis of LDs in DCs can affect the ability of T cells to initiate an antitumor response.^234^

In tumor cells, the activation of intracellular signaling pathways enhances FA synthesis and LD accumulation, leading to senescence in effector T cells.^235^ These senescent T cells, characterized by dysfunction within the tumor microenvironment, exhibit changes in lipid species composition and an accumulation of LDs. This accumulation is correlated with the increased activity of group IVA phospholipase A2. Inhibiting the activity of this enzyme has been shown to reverse T-cell senescence, resulting in reduced tumor size and prolonged survival in tumor-bearing mice.^31^

While T cells have traditionally been the focus, macrophages have recently garnered increased attention. Agonists for TLRs, such as TLR2, TLR3, TLR4, and TLR7, have been found to increase LD counts and increase the expression of key proteins involved in LD biogenesis, such as perilipin 2 and DGAT2, in both thioglycollate-elicited peritoneal mouse macrophages and human monocyte-derived macrophages.^236^ In addition, TLR2 is crucial for stimulating LD formation in macrophages in response to infections by pathogens such as *Trypanosoma cruzi*, *Mycobacterium bovis* BCG, and *Histoplasma capsulatum*.^237,238^ These findings suggest that LD formation in myeloid cells is a consequence of host defense mechanisms orchestrated by pattern recognition receptor activation.

Adipose triglyceride lipase (ATGL) is recognized as a critical enzyme for triglyceride breakdown within cell LDs. Inhibition of ATGL-mediated lipolysis in macrophages enhances lipid accumulation and hampers the production of the cytokine IL-6.^239,240^ In ATGL-null macrophages, increased FA uptake attempts to compensate for reduced lipolysis, which is crucial for ATP synthesis and effective phagocytosis.^241^ Therefore, targeting enzymes such as ATGL, which are involved in lipid metabolism, has therapeutic potential for diseases characterized by dysregulated lipid metabolism and inflammation, including atherosclerosis and metabolic disorders.^239,240^

LDs are also crucial for DC function, particularly in T cell activation. In granulocyte‒macrophage colony‒stimulating factor (GM-CSF)-induced bone marrow-derived dendritic cells (BMDCs), LD accumulation is correlated with increased expression of IFN-γ-induced GTPase (IGTP).^242^ GM-CSF-derived BMDCs lacking IGTP fail to accumulate lipid droplets and exhibit defects in antigen cross-presentation and CD8^+^ T cell activation. IGTP interacts with adipocyte differentiation-related proteins (also known as perilipin-2) on LDs, preventing phagosome degradation and promoting immune function.^242^ Additionally, changes in LD composition can affect DC immune function. For example, tumor-derived factors alter the lipid composition of LDs and suppress DC responses to CD8^+^ T cells.^243^

In summary, different immune cells rely on distinct lipid metabolic environments to fulfill their functional needs. M1 macrophages enhance the immune response through glycolysis and lipid synthesis, upregulating ACLY, ACC, and FASN to generate proinflammatory lipid molecules such as prostaglandins while promoting neutral lipid storage *via* perilipin 2. In contrast, M2 macrophages rely on FAO to maintain anti-inflammatory functions, with high activity of CPT1 and acyl-CoA dehydrogenases and lower levels of lipid synthesis and LD formation.^92^ Activated T cells upregulate ACLY, FASN, SREBP2, and 3-hydroxy-3-methylglutaryl-coA reductase (HMGCR) to increase lipid and cholesterol metabolism, supporting membrane phospholipid synthesis and immunological synapse formation, primarily through glycolysis, although FAO is crucial for their memory function.^35^ Additionally, activated T cells form limited LDs, with lipids primarily used for membrane expansion rather than storage.^35^ Activated B cells upregulate FASN and cholesterol synthesis to support proliferation, endoplasmic reticulum expansion, and antibody secretion, potentially supplementing metabolic demands through increased FAO. However, B cells have limited LD formation capacity, weak perilipin 2 expression, and low lipid storage levels.^244^ Neutrophils require FASN for survival and differentiation, with FAO supporting energy production during differentiation. Disruption of lipid metabolism or FAO impairs neutrophil function and recruitment to infection sites.^207,208^ DCs rely on FASN and FAO for maturation and antigen presentation. Inhibition of these pathways impairs DC function and antitumor immunity. Lipid droplets in DCs are important for T cell activation, and their composition can affect immune responses.^209,230^ Overall, lipid metabolism, including FASN, FAO, and LDs, plays a key role in immune cell differentiation and function, with potential therapeutic implications for immune modulation.

## Regulatory mechanisms of lipid metabolism in immune cell responses

Immune cells rely on lipids for membrane structure, energy, and signaling, making lipid metabolism essential for immune responses.^245^ This process is tightly regulated at multiple levels, including genetic, epigenetic, posttranscriptional, and posttranslational levels, ensuring that lipid profiles are dynamically adjusted to meet the metabolic demands of immune cell activation and inflammatory responses.^246–249^ Disruption of these regulatory mechanisms can contribute to various diseases, such as type 2 diabetes mellitus (T2DM) and fatty liver disease, especially cancer. This chapter explores the different regulatory mechanisms involved in lipid metabolism within immune cells, with a focus on genetic regulation, epigenetic and posttranscriptional modulation, and PTMs.

### Genetic regulation

Lipid metabolism in immune cells is governed by a variety of genetic regulators, including transcription factors, nuclear receptors, and enzymes that control lipid biosynthesis, oxidation, and storage. These genetic regulators ensure that immune cells can adapt to various environmental cues and immune stimuli by altering lipid metabolic pathways accordingly.

One key regulatory factor is the peroxisome proliferator-activated receptor (PPAR) family, particularly PPAR-γ and PPAR-α (Fig. 6). PPAR-γ is a ligand-activated nuclear receptor that is expressed primarily in macrophages, where it regulates FA uptake and storage while also modulating inflammation and promoting immune tolerance.^250–252^ PPAR-γ exerts significant anti-inflammatory effects by regulating triglyceride metabolism, lipid uptake, cholesterol efflux, and macrophage polarization and inhibiting inflammatory signaling pathways.^250^ Furthermore, through posttranslational modifications (PTMs), PPAR-γ modulates its interactions with transcriptional ligands and coactivators or corepressors, thereby influencing the regulation of downstream target genes.^250^ For example, studies have shown that PTMs of PPAR-γ regulate lipid synthesis in response to wound microenvironment cues and that metabolic reprogramming coordinates the function of reparative macrophages.^253^ Additionally, PPAR-γ is a required transcription factor for FAO induction in the tumor-associated macrophage (TAM) polarization process, which promotes tumor growth.^254^

PPAR-α is essential for T cells, where it promotes FAO and provides the energy required for immune cell activation and function. For example, selective activation of PPAR-α enhances free FA metabolism and downregulates the gene expression of *Il17a* and *Il23r*, thereby suppressing the metabolic program of Th17 cells. This mechanism may represent a viable therapeutic option for autoimmune diseases.^255^ Furthermore, fenofibrate (a synthetic ligand of PPAR-α) regulates Th1/Th17/Treg cell responses by activating PPAR-α/LXR-β signaling.^256^

The immunogenicity of DCs is regulated by PPAR-γ. The activation of PPAR-γ in DCs inhibits the expression of EBI1 ligand chemokines and CCR7, both of which play key roles in DC migration to lymph nodes.^257^ Studies have also shown that the number of eosinophils is increased in PPAR-α-deficient mice, whereas the activation of PPAR-γ can reduce the number of eosinophils. In addition, the activation of both PPAR-α and PPAR-γ not only inhibits eosinophil chemotaxis but also diminishes their antibody-dependent cellular cytotoxicity.^258^

Another critical genetic regulator is SREBP, which responds to cellular lipid levels and regulates the expression of genes involved in lipid synthesis (Fig. 6).^259,260^ Studies have shown that hepatic SREBP signaling mediates circadian communication within the liver and that lipidomic changes dependent on SREBP cleavage-activating protein (SCAP) lead to increased metabolic rhythmicity in liver macrophages.^261^ In the absence of SCAP-mediated activation of SREBP1a, increased M1 macrophage polarization results in reduced cholesterol efflux downstream of 25-hydroxycholesterol (HC)-dependent LXRα activation.^262^ Moreover, mouse macrophages lacking SREBP1a, when exposed to bacterial LPS triggering TLR4 activation, exhibit impaired lipogenesis, resulting in compromised innate inflammatory responses marked by reduced cytokine production.^263^ SREBP1a promotes the biosynthesis of anti-inflammatory FAs by regulating lipid metabolism in macrophages, thereby inhibiting inflammation.^264^

For T cells, during viral infections, SREBPs are pivotal in regulating CD8^+^ T cells function through initiating lipogenesis to support membrane synthesis.^265,266^ Additionally, mammalian target of rapamycin complex 1 (mTORC1)-raptor activates lipogenic pathways *via* SREBP1c, increasing cell proliferation and facilitating Th2 cell differentiation.^267^ In addition to Th2 cells, Th17 cells depend on ACC-mediated lipogenesis for phospholipid production in their plasma membranes, a process that suppresses Th17 cell development while promoting Treg formation.^268,269^ Additionally, SREBP activity is reportedly upregulated in tumor-infiltrating Tregs, and blocking SREBP signaling disrupts the activation of PI3K in these cells, further suggesting that lipid signaling enhances the functional specialization of Tregs in tumors.^203^

Moreover, metabolic reprogramming in activated B cells requires SREBP signaling. SREBP signaling in B cells is crucial for antibody responses and the generation of GCs, memory B cells, and bone marrow plasma cells. Under mitogen stimulation, SCAP-deficient B cells fail to proliferate, and lipid rafts are reduced.^270^ These genetic regulators collectively fine-tune lipid metabolism in immune cells, providing essential support for their immune functions while also offering potential targets for therapeutic intervention in immune-related diseases. Currently, the regulatory mechanisms of lipid metabolism in immune cells are not fully understood, and research faces challenges such as model limitations, the complexity of lipid metabolic pathways, difficulties in drug target development, and individual variability. Future studies need to explore these mechanisms in greater depth and overcome these obstacles to advance the application of lipid metabolism regulation in the treatment of immune-related diseases.

### Epigenetic and posttranscriptional regulation

In addition to genetic regulation, lipid metabolism in immune cells is also controlled by epigenetic modifications and posttranscriptional mechanisms, providing further layers of fine-tuning. These regulatory processes do not alter the DNA sequence but instead modify gene expression through mechanisms such as DNA methylation, histone modifications, and the action of noncoding RNAs.

DNA methylation is an important epigenetic modification that influences lipid metabolism by repressing the expression of certain genes, particularly through the inhibition of transcription factor activation. The selective activation of adipose tissue macrophages is controlled by DNA methylation at the PPAR-γ promoter. DNA methylation at the PPAR-γ promoter blocks the alternative activation of macrophages, whereas high levels of DNA methylation promote inflammatory responses and insulin resistance.^271^ Furthermore, studies have shown that histone modifications at the PPAR-γ promoter can influence the production of cytokines and autoantibodies. For example, the deletion of HDAC9 in mouse CD4^+^ T cells increases histone H3 lysine 9 acetylation (H3K9ac) and H3K18ac at the PPAR-γ promoter, promoting a shift in T cell cytokine production toward a more anti-inflammatory profile while reducing the production of anti-dsDNA autoantibodies by B cells.^272^

Studies have shown that targeting methylation-related genes and proteins, such as DNA methyltransferase 3beta and TET methylcytosine dioxygenase 3 (TET3), can increase DNA methylation, thereby inhibiting PMA-induced NETosis.^273^ During the differentiation of DCs from monocytes, the expression of DC-SIGN (CD209) is linked to a reduction in DNA methylation.^274^ Treatment of monocyte-derived DCs with the methyltransferase inhibitor 5-azacytidine increases the levels of the costimulatory molecules CD40 and CD86, which, in turn, induce activated T cells to express the cytokines IFN-γ and IL-17A.^274^

Noncoding RNAs, particularly microRNAs, are vital regulators of lipid metabolism. microRNAs can bind to mRNA transcripts and inhibit their translation or promote their degradation, thus modulating lipid metabolic pathways. For example, miR-33 is an intronic microRNA within the gene encoding the SREBP2 transcription factor. The inhibition of miR-33 has been shown to promote cholesterol efflux in macrophages by targeting the cholesterol transporter ATP-binding cassette subfamily A member 1 (ABCA1), thereby reducing the burden of atherosclerotic plaques.^275^ In addition, the overexpression of the long noncoding RNA homeobox transcript antisense intergenic RNA (HOTAIR) can effectively reduce lipid uptake and suppress immune responses by downregulating the expression of TNF-α and IL-6 during foam cell formation. Mechanistically, HOTAIR alleviates foam cell formation by inhibiting the expression of miR-19a-3p.^276^ Other studies have shown that oxidized LDL induces the expression and release of miR-155 in macrophages and that miR-155 is essential for mediating oxidized LDL-induced lipid uptake and ROS production in macrophages.^277^ Reducing miR-155-5p levels and subsequently increasing the expression of *LXRα* leads to increased ABCA1- and ABCG1-dependent cholesterol efflux, which promotes macrophage polarization to the M2 phenotype.^278^

MiR-223 controls neutrophil function by inhibiting the transcription factor myocyte-specific enhancer factor 2 C (MEF2C). In mice, the absence of miR-223 results in a twofold increase in hyperreactive neutrophils, promoting acute inflammation in multiple organs through the involvement of myeloperoxidase and ROS.^279^ Furthermore, miR-130a is implicated in neutrophil development. The overexpression of miR-130a downregulates CCAAT/enhancer binding protein-ε (C/EBP-ε), reducing the synthesis of secondary granule proteins such as lactoferrin, cathelicidin, and lipocalin-2, which leads to the differentiation of neutrophils with an immature phenotype.^279^

Various microRNAs play important roles in the differentiation, activation, and functional regulation of DCs. For example, miR-144/451 directly targets interferon regulatory factor 5 (IRF5), inhibiting its expression and reducing DC activation.^280^ In contrast, miR-148a targets MAF bZIP transcription factor B, promoting the differentiation of monocyte-derived DCs.^281^ Additionally, miR-9 is upregulated in bone marrow-derived DCs and conventional DC1s, promoting DC activation and enhancing their ability to stimulate T cells.^282^

microRNAs play crucial roles in regulating mast cell differentiation, proliferation, survival, apoptosis, stress responses, effector functions, and the resolution of immune responses.^283^ For example, miR-210 and miR-221-3p participate in the pathogenesis of asthma by promoting mast cell activation and Th2 cytokine production. In contrast, miR-143 targets IL-13Rα1 to reduce mast cell activation and subsequent allergic reactions. More detailed information can be found in the referenced study.^283^

These epigenetic and posttranscriptional regulatory mechanisms add another layer of complexity to lipid metabolism, allowing immune cells to adapt to environmental stimuli and modulate their function in response to immune challenges.

### Posttranslational regulation

PTMs are another crucial aspect of regulating lipid metabolism in immune cells. PTMs such as phosphorylation and ubiquitination control the activity, stability, and interactions of lipid-metabolizing enzymes, enabling immune cells to rapidly adjust their metabolic pathways in response to immune activation.

Phosphorylation plays a central role in lipid metabolism regulation. AMPK, an energy-sensing enzyme, regulates lipid metabolism by phosphorylating key enzymes involved in FAO and synthesis. Research has shown that the accumulated 25-HC in lysosomes competes with cholesterol for binding to GPR155, inhibiting the kinase mTORC1, which leads to the activation of AMPKα and metabolic reprogramming. AMPKα also phosphorylates STAT6 at Ser564 to increase STAT6 activation and arginase 1 production, suggesting that cholesterol 25-hydroxylase acts as an immune metabolic checkpoint that can manipulate macrophage fate to reshape CD8^+^ T-cell surveillance and antitumor responses.^284,285^ Additionally, activation of the ROS-AMPK-mTORC1-autophagy pathway enhances M1-to-M2 polarization, cholesterol efflux, and the anti-inflammatory response both in vitro and in vivo in murine bone marrow-derived M1 macrophage (BMDM1) cells.^286^ Notably, AMPK activation also increases mitochondrial FAO in activated CD4^+^ T cells, promoting natural Treg cell differentiation. Furthermore, AMPK agonists promote FAO and natural Treg differentiation *via* β1-adrenergic receptor signaling.^287^

Ubiquitination regulates lipid metabolism by targeting enzymes for degradation, thereby controlling their levels and ensuring the balance of lipid metabolic processes during immune activation to prevent metabolic dysregulation. For example, the known protein phosphatase protein tyrosine phosphatase B (PtpB) from *Mycobacterium tuberculosis* dephosphorylates phosphatidylinositol-4-phosphate and phosphatidylinositol-(4,5)-bisphosphate in host cell membranes, which disrupts the membrane localization of cleaved gasdermin D (GSDMD), thereby inhibiting cytokine release and pyroptosis in macrophages. This phosphatase activity requires PtpB to bind to ubiquitin.^288^ Disruption of phosphatase activity or the ubiquitin-binding motif of PtpB can enhance host GSDMD-dependent immune responses and reduce the survival of intracellular pathogens.

Moreover, the inhibition of RAD18 E3 ubiquitin ligase-mediated ubiquitination leads to the stabilization and upregulation of Ewing’s sarcoma RNA-binding protein 1, which in turn increases the expression of PPAR-α and FABP1. This causes T cell dysfunction and the malignant progression of hepatocellular carcinoma.^289^ In B cells, PPAR-γ, an E3 ubiquitin ligase, impedes the stability of phosphorylated Stat6 and promotes the inhibition of PGE_2_-mediated asthma-associated IgE production.^47,290^ In neutrophils, Toll/interleukin-1 receptor domain-containing protein C (TcpC) is an E3 ubiquitin ligase that targets peptidyl arginine deiminase 4 (PAD4), inhibiting the formation of NETs by enhancing the ubiquitination-mediated degradation of PAD4.^291^ Recent studies have demonstrated that ubiquitin enzymes, including E3 ubiquitin ligases and deubiquitinases, are key regulators of DC-mediated immune functions.^292^ For example, DCs with defective MHC-II ubiquitination fail to present antigens effectively.^293^ Additionally, ubiquitination-induced downregulation of MHC-II is essential for the migration of CD206^+^ monocyte-derived DCs to skin-draining lymph nodes (sdLNs). CD206^+^ monocyte-derived DCs from membrane-associated ring-CH-type finger 1 knockout mice exhibit overexpression of MHC-II, reduced expression of IRF4 and CCR7, and impaired migration from the skin to the sdLN.^294^

In addition to these traditional PTMs, N6-methyladenosine (m6A) RNA modification has emerged as an important mechanism of posttranscriptional regulation that influences lipid metabolism in immune cells. Although m6A modification does not directly modify proteins, it indirectly regulates protein expression by modulating RNA, particularly the stability, translation efficiency, and splicing of mRNAs, thereby affecting the levels and activity of lipid-metabolizing enzymes. For example, lactate-mediated m6A modification of *tribble homolog 1* (*Trib1*) mRNA through methyltransferase-like 3 (METTL3) promotes M2 macrophage polarization, increasing its stability.^295^

Numerous studies have shown that m6A regulates T cell functions through various mechanisms, with key regulatory factors such as m6A itself, METTL3, and Wilms tumor 1-associated protein (Wtap) proteins controlling T cell homeostasis, including development, activation, survival, and T cell exhaustion.^296^ Inhibitory factors such as the suppressor of cytokine signaling (SOCS) gene family or the stabilization of mRNAs for Ca^2+^ release-activated Ca^2+^ modulator 1 (Orai1) and receptor-interacting serine/threonine-protein kinase 1 (Ripk1), which are marked by m6A, contribute to the regulation of T cell proliferation.^297,298^

Although research on m6A modifications in B cells is relatively limited, studies have shown that m6A methylation and its reader proteins play critical regulatory roles in early B-cell development. The inhibition of m6A methylation severely impairs B cell development in mice.^299^ Additionally, m6A methylation mediated by METTL14 is essential for the response of GC B cells in mice. Deletion of METTL14 in B cells leads to impaired GC B cell proliferation and defects in antibody responses.^300^ We speculate that targeting m6A modifications has great potential for improving adoptive cell therapy. Although attempts to regulate m6A methylation in chimeric antigen receptor-modified (CAR) T and B cells have not yet been reported, considering the critical role of m6A regulators in determining T and B cell function and fate, new therapeutic strategies are expected to emerge.

m6A modification plays crucial roles in the activation, migration, and immune response of neutrophils. METTL3 regulates the mRNA stability of TLR4, enhancing its translation and promoting the activation of TLR4 signaling, which drives neutrophil activation and function.^301^ m6A modification also regulates the surface expression of C-X-C motif receptor 2 (CXCR2), controlling the release of neutrophils from the bone marrow into the peripheral blood.^301,302^ Additionally, the m6A demethylase alkylation repair homolog protein 5 (ALKBH5) plays a significant role in regulating neutrophil migration. ALKBH5 removes m6A modifications from *granulocyte colony-stimulating factor receptor* (*G-csfr*) mRNA, increasing the stability of *G-csfr* mRNA, which in turn promotes the mobilization and generation of neutrophils.^303^

Through these PTMs, immune cells can dynamically adjust their lipid metabolic pathways to meet the demands of immune responses, ensuring that immune cells remain metabolically flexible and functionally responsive during activation.

## The role of lipid metabolism in diseases

Understanding the regulation of lipid metabolism in the immune cell response under normal conditions can shed light on how disruptions in lipid metabolism can skew immune cells toward the immune response. This insight paves the way for exploring lipid metabolism as an effective target for treating various diseases. Below, we elucidate various types of diseases, including autoimmune diseases, cancer, neurodegenerative diseases (NDDs), cardiovascular diseases (CVDs), aging and metabolic disorders, which may be influenced by lipid metabolism (Fig. 1).

### Lipid metabolism in autoimmune diseases

Chronic and uncontrolled immune responses lead to inflammatory reactions, resulting in autoimmune diseases that are difficult to treat and impose a heavy burden on patients.^304^ Emerging evidence in recent decades suggests that aberrant lipid metabolism plays a key role in the pathological processes of various autoimmune diseases.^24,305^ FAs are strongly associated with autoimmunity and can mediate the development of autoimmune diseases by regulating the polarization, differentiation, and activity of immune cells.^306^

The level of butyrate, a SCFA, is strongly related to the onset of type 1 diabetes mellitus (T1DM), and dietary interventions that maintain optimal levels of butyrate may significantly reduce the risk of developing T1DM.^307^ Accumulated evidence has shown that feeding nonobese diabetic mice diets enriched with acetate and butyrate can prevent T1DM.^308^ Conversely, reduced dietary fiber intake in mice leads to lower SCFA levels, disrupting homeostasis and impairing specific antibody functions, thus increasing susceptibility to infections.^44^ In addition, butyrate not only enhances histone H3 acetylation in the promoter and conserved noncoding sequence region of the Foxp3 motif by inducing the differentiation of Treg cells in vivo but also induces apoptosis in proinflammatory T cells and inhibits IFN-γ secretion in colonic epithelial cells, thereby ameliorating colitis progression.^309^

SCFAs also strengthen the tight junctions of intestinal epithelial cells and lower inflammatory factor levels to maintain intestinal immune homeostasis. This action slows the progression of IBD *via* GPR-mediated immune signaling cascades and reinstates tight junction proteins, including zonula occludens-1, occludin and epithelial cadherin.^310^ SCFAs have also been shown to attenuate systemic lupus erythematosus (SLE) and renal lesions in female lupus-susceptible mice, prolonging their survival by affecting B-cell-intrinsic epigenetic mechanisms and modulating autoantibody responses.^311^

However, SCFAs have a bidirectional effect on neutrophil-mediated therapy for autoimmune diseases. Oral administration of acetate induces neutrophil chemotaxis in vivo *via* GPR43, which may promote the resolution of IBD and arthritis.^312^ In contrast, propionate and butyrate enhance the expression of L-selectin on the surface of neutrophils, facilitating their migration to inflammatory sites, a process that contributes to the progression of inflammatory responses.^61^ Moreover, butyrate can induce intercellular adhesion molecule-1 (ICAM-1) and E-selectin, which may have beneficial or detrimental effects depending on the pathophysiological process involved.^313^

n-3 PUFAs also mediate autoimmune disease progression. Supplementation with n-3 PUFAs regulates T cell function and suppresses circulating levels of inflammatory cytokines, thus alleviating T cell-mediated autoimmunity.^314^ Elevated endogenous n-3 PUFAs inhibit Th17 cell function, maintain Treg cell populations, and alleviate lesions in a 3D psoriatic skin model.^315^ These findings underscore the therapeutic potential of n-3 PUFAs for psoriasis treatment. Similarly, dietary intake of n-3 PUFAs markedly reduces the incidence of T1DM by interfering with CD4^+^ T cell differentiation through inhibition of the mTORC1 pathway and decreasing the expression of IFN-γ, TNF-α and IL-17.^316^ In colitis, n-3 PUFAs significantly upregulate claudin-1 and occludin and downregulate the expression of IL-6, IFN-γ and TNF-α by regulating cellular transduction pathways (e.g., the NLRP3/IL-1β, IL-6/STAT3, and wingless-related integration site (Wnt)/β-catenin pathways), thereby maintaining intestinal barrier function and attenuating intestinal injury and necrosis.^317–319^

Genetic variations in lipid-metabolizing enzymes and transporters, particularly those affecting lipid metabolites, play crucial roles in regulating immune cell function and are key in the pathogenesis of autoimmune diseases. For example, loss-of-function mutations in protein tyrosine phosphatase, nonreceptor type 2 (PTPN2) are associated with an increased risk of IBD and RA, with PTPN2’s pathogenic variant amplifying the link between intestinal inflammation and arthritis by converting colonic Tregs to exTregs.^320^ Furthermore, genetic variations in LDL metabolism-related genes, such as mutations in the *apolipoprotein B* (*APOB)* gene, have been linked to a lower risk of RA, possibly through their inhibition of proinflammatory cytokine production.^321^

Similarly, orosomucoid-like (ORMDL) proteins, which mediate feedback inhibition of de novo sphingolipid synthesis, are implicated in inflammation and the progression of autoimmune diseases. Studies have shown that the deletion of ORMDL1 and ORMDL3 in mice disrupts blood homeostasis and reduces immune cell content in the peripheral blood and spleen.^322^ Such genetic variations not only affect lipid metabolism and distribution but also influence the strength and direction of immune responses, potentially promoting chronic inflammation and immune dysregulation, thus increasing susceptibility to autoimmune diseases. Further research is needed to elucidate the complex relationship between lipid metabolism and immune responses, providing new targets and strategies for the early diagnosis, prevention, and personalized treatment of autoimmune diseases.

### Lipid metabolism in cancer

Tumors undergo metabolic transformations that enable unchecked proliferation and gradual spread throughout the body, endangering human health. Abnormal lipid metabolism influences cancer progression, as increased synthesis or uptake of lipids accelerates the rapid proliferation and metastasis of cancer cells.^323^ Therefore, targeting lipid metabolism in cancer has become an effective strategy for developing antitumor drugs.

The increased synthesis of FAs is a well-documented metabolic alteration in cancer. FASN catalyzes FA synthesis, and its overexpression is closely linked to malignant tumor progression.^324^ FASN overexpression has been shown to promote epithelial‒mesenchymal transition (EMT), which increases the metastatic capacity and tumor load of cancer cells, leading to peritoneal metastasis in ovarian cancer.^325^ Additionally, FASN inhibition can attenuate the palmitoylation of Wnt in tumor cells and impede the Wnt/β-catenin pathway, which can reduce cancer cell metastasis and attenuate colorectal cancer (CRC) and prostate cancer.^326,327^

Polymorphonuclear myeloid-derived suppressor cells (PMN-MDSCs) are a class of pathologically activated neutrophils with significant immunosuppressive functions. As key components of the tumor microenvironment, they promote tumor immune tolerance and contribute to the failure of tumor immunotherapy.^328^ Fatty acid transport protein 2 (FATP2) reportedly mediates and inhibits the activity of PMN-MDSCs, which significantly delays tumor progression.^329^ The underlying mechanism primarily involves the uptake of arachidonic acid and the synthesis of PGE_2_, a process crucial for the expression of proinflammatory genes by PMN-MDSCs. Furthermore, two additional studies demonstrated the existence of a PGE_2_-mediated feedback loop between FATP2 and receptor-interacting protein kinase 3, which significantly enhances the suppression of CD8 T cell function by PMN-MDSCs.^330,331^ Therefore, the selective inhibition of PMN-MDSCs by FATP2 presents a novel therapeutic target for cancer treatment.

N-3 PUFAs are well known for their anti-inflammatory properties, whereas n-6 PUFAs serve as precursors to proinflammatory molecules.^23^ N-3 PUFAs can exert antitumor effects by inhibiting cancer cell proliferation, promoting apoptosis, reducing metastasis and suppressing inflammatory responses through the activation of PPAR-γ.^332^ Furthermore, EPA and DHA are metabolized into corresponding ethanolamine derivatives in cancer cells to induce anticancer effects, and they have been investigated as potential dietary agents for the prevention of breast cancer.^333^ DHA inhibits granzyme B expression and the capacity of CRC cells to undergo EMT and invasion, highlighting the underlying mechanisms of the anticancer activity of DHA.^334^ Interestingly, lipid mediators produced by EPA and DHA, including resolvins, protectins, and maresins, are involved in modulating inflammatory pathways and mediating cancer progression.^335^

Cholesterol regulates cancer-associated cell signaling pathways, such as the Wnt/frizzled (Fzd), PI3K/AKT and p53 pathways, whose synthesis and transport are critical factors in cancer development and are highly important in tumorigenesis, progression and metastasis.^336^ The deletion or mutation of *p53*, a tumor suppressor gene, increases the expression of mevalonate pathway genes, leading to aberrant cholesterol synthesis, which may have sufficient oncogenic potential.^337^ In addition, the deletion of the tumor suppressor phosphatase and tensin homolog (PTEN) and stimulation of the PI3K/AKT pathway lead to abnormal cholesterol accumulation, promoting prostate cancer proliferation and invasion.^338^

Cholesterol is a multifaceted metabolite known to modulate various processes in cancer.^339^ Mechanistically, cholesterol and its metabolites affect cancer cells by altering immune reactions such as cell enrichment, autophagy and ferroptosis.^336^ The cholesterol metabolite 27-HC, a selective estrogen receptor modulator and LXR agonist, promotes cancer metastasis and contributes to the growth of ER^+^ breast tumors by acting directly on neutrophils and indirectly on γδ-T cells, as well as by enhancing the resistance of metastatic cells to ferroptosis.^340^ Consistent with these findings, chronic exposure of cells to 27-HC leads to increased cellular uptake and/or cholesterol biosynthesis, promoting tumor metastasis.^340^ Notably, the inhibition or elimination of the 27-HC biosynthetic enzyme CYP27A1 effectively reduces cancer metastasis. In recent years, the enrichment of TAMs has been demonstrated to impact tumor growth, invasion, and metastasis. During tumor progression, tumor cells release factors that facilitate membrane cholesterol efflux from TAMs, driving TAM reprogramming and promoting tumor growth; however, the deletion of ATP-binding cassette (ABC) transporter proteins can block TAM reprogramming and inhibit tumor growth.^341^

FA metabolism plays a crucial role in the occurrence and progression of CRC. Studies have shown that SCD, the rate-limiting enzyme in the biosynthesis of unsaturated FAs, is expressed at low levels in peritoneal metastasis and is associated with poor prognosis in CRC patients.^342^ Additionally, FASN, a key enzyme in de novo FA synthesis, is involved in regulating body weight and tumor growth. Genetic polymorphisms in FASN are significantly associated with the risk of prostate cancer. In particular, inhibiting FASN may reduce prostate cancer-specific mortality, especially in overweight men.^28,343^ Furthermore, FASN polymorphisms have been shown to predict the treatment outcomes of metastatic CRC patients receiving bevacizumab, suggesting that lipid metabolism pathways may play a role in resistance to anti-VEGF therapy.^344^

*ApoE* gene variations are also associated with the development and progression of brain tumors. Carriers of the ApoE ε4 allele, in particular, are more likely to experience cognitive impairments such as memory and executive dysfunction in brain cancer patients. Other single nucleotide polymorphisms in the *ApoE* gene have been linked to cognitive outcomes, indicating that ApoE may play a significant role in the occurrence and clinical prognosis of brain tumors.^345^ Overall, genetic variations in lipid-metabolizing enzymes and transporters not only alter lipid metabolism and distribution but also affect cell membrane composition and function, as well as signal transduction and immune responses, thereby influencing cancer initiation, progression, and metastasis. Further research into the relationship between lipid metabolism and cancer will not only help elucidate the molecular mechanisms of tumorigenesis but also provide new potential targets for early diagnosis, prevention, and personalized treatment of cancer.^346^

### Lipid metabolism in neurodegenerative diseases

With the increase in the global population, NDDs are increasingly acknowledged as significant contributors to morbidity and mortality.^347^ Abnormal lipid metabolism can affect normal brain development and function, as well as the survival, function, and communication of nerve cells, thereby promoting or exacerbating the development of NDDs, suggesting the importance of lipids and their metabolites in NDDs.^348^

Alzheimer’s disease (AD) and Parkinson’s disease (PD) are influenced by SCFAs, which can affect psychological functions, including emotions and cognitive processes, by crossing the blood‒brain barrier (BBB) and influencing brain metabolism.^349^ For example, valproic acid reduces amyloid-beta (Aβ) and alpha-synuclein aggregation by inhibiting HDAC activity, thereby reducing the number of neuroinflammatory plaques and improving memory deficits.^350^ SCFAs also play a role in regulating T cell differentiation to directly impact the inflammatory response within the central nervous system. For example, SCFAs can induce Foxp3 expression, promote Treg cell differentiation, improve EAE lesions, and reduce damage.^351^ Moreover, SCFAs can interfere with NDDs by modulating inflammatory factors and microglia. Acetic acid and butyrate decrease the mRNA levels of microglial inflammatory signaling molecules, including *IL-1β, IL-6*, and *TNF-α*, while increasing the mRNA levels of *TGF-β1* and *IL-4*, suggesting that acetic acid can ameliorate NDD lesions.^352,353^

Similarly, LCFAs also influence the development of NDDs, and n-3 PUFAs are important for maintaining brain development and function. A deficiency in n-3 PUFAs can lead to neuroinflammation, cognitive impairments, suboptimal neurogenesis, neurotransmitter metabolism defects, and impaired brain growth and development, thereby affecting the progression of NDDs such as AD.^354,355^ In addition, n-3 PUFAs have significant protective effects against inflammatory damage to neurons and glial cells, enhancing myelin sheath and microglial phagocytosis and reducing inflammation to improve the brain environment.^356^ DHA, an important member of the n-3 PUFA family, is highly concentrated across all brain regions and is closely associated with the progression of NDDs.^355^ Furthermore, DHA provides neuroprotection by reducing Aβ deposition in AD mouse models.^357^ Dietary administration of DHA can lower lipid peroxides and ROS in cortical hippocampal tissues, improving cognitive impairments.^355^

Disruption of cholesterol metabolism in the brain is correlated with NDDs.^358^ The brain, which contains approximately 25% of the body’s total cholesterol, primarily stores cholesterol in the myelin sheath, astrocytes, and neuronal cell membranes.^359^ Brain cholesterol is believed to be crucial for maintaining cell morphology, neuronal transmission, and synaptic formation.^360^ The involvement of cholesterol in the pathogenesis of NDDs is correlated with ApoE.^361^ Cholesterol is synthesized in astrocytes and transported to neurons *via* ApoE, which is essential for maintaining brain signal transmission and basic functions.^362^ Deficiency in ApoE, the primary apolipoprotein of high-density lipoprotein (HDL) in the central nervous system, affects brain development and cause cognitive impairments.^363^ In contrast, HDL, which is rich in ApoE, alleviates AD pathology by reducing Aβ deposition and inflammation in blood vessels.^364^ These observations indicate an intensive link between lipid metabolism and the pathogenesis of NDDs. Preventing or slowing the onset of diseases through effective intervention in lipid metabolism has become a promising target.

Genetic variations in lipid metabolism enzymes can have a profound impact on brain health. For example, different alleles of the *ApoE* gene, such as ε2 and ε4, have been shown to be closely associated with susceptibility to MS. Specifically, homozygosity for the APOE ε4 allele is linked to cognitive decline in patients with relapsing–remitting MS.^365^ Additionally, sphingolipid metabolism in neurons is crucial for brain health. Genetic variations in genes and proteins related to sphingolipid metabolism have been implicated in various NDDs. For example, *ORMDL3* mRNA expression is significantly upregulated in serum samples from AD patients. Studies suggest that inhibiting ORMDL3 function could prevent the onset of AD.^366^ Furthermore, genetic polymorphisms in cholesteryl ester transfer protein (CETP), particularly homozygosity for the CETP V405 valine allele, have been shown to delay memory decline and reduce the risk of dementia and AD.^367,368^ In contrast, the CETP I405V polymorphism is associated with faster cognitive decline and an increased risk of AD.^367,368^ In summary, screening for individual lipid metabolism gene variations could help design personalized prevention and treatment approaches, thereby more effectively addressing the challenges of NDDs.

### Lipid metabolism in cardiovascular diseases

CVDs are major contributors to global morbidity and mortality, with atherosclerosis-induced ischemic heart disease and stroke being the primary causes of death.^369,370^ Lipid metabolism products play a key role in CVD, as they mediate immune responses and affect the functionality of leukocytes, blood vessels, and heart cells, thus influencing the vasculature and heart.^371^ Therefore, understanding the function and mechanism of lipid metabolism in the pathogenesis of CVDs probably opens new avenues for preventing and treating these conditions.

FAs influence the occurrence and development of CVDs, with SCFAs playing an active role. Endothelial dysfunction leads to impaired barrier integrity and triggers the production of proinflammatory cytokines, chemokines, and ROS, along with the recruitment, adherence, and subendothelial migration of proinflammatory leukocytes, resulting in atherosclerotic lesions.^372^ However, butyrate and propionate reduce the expression of vascular cell adhesion molecule-1 (VCAM-1), which may prevent or treat CVDs by attenuating endothelial activation.^373^ Additionally, propionate and butyrate play crucial roles in atherosclerosis management by regulating Treg cell production and suppressing HDACs.^117^ These findings imply that SCFAs can mitigate CVD progression through anti-inflammatory and metabolic modulation.

N-3 PUFAs can lower plasma triglycerides, the resting heart rate, and blood pressure; improve myocardial filling and efficiency; reduce inflammation; and enhance vascular function.^374^ In the short term, n-3 PUFA consumption increases nitric oxide (NO) production, mitigates vasoconstrictive responses to norepinephrine and angiotensin II, enhances vasodilatory responses, and improves arterial compliance.^375^ Furthermore, the consumption of n-3 PUFAs attenuates the levels of VCAM-1, ICAM-1, and E-selectin, reducing key leukocyte‒endothelial interactions and contributing to a reduction in CVD incidence.^376^

Plasma levels of cholesterol, LDL cholesterol, and apolipoproteins strongly correlate with clinical atherosclerosis and other CVDs.^377,378^ Studies underscore that oxidized LDL accelerates atherosclerotic plaque development by triggering endothelial cell dysfunction, facilitating macrophage foam cell formation, and stimulating smooth muscle cell migration and proliferation.^379^ In addition, oxidized LDL has proinflammatory properties and promotes thrombosis by activating endothelial cells, smooth muscle cells, macrophages, and platelets.^380^ The inability to effectively clear LDL from the circulation can lead to large atherosclerotic lesions containing macrophage foam cells.^381^ This may be caused by dysfunction of the transcription factor EB-P300-bromodomain-containing protein 4 axis.^382^ Moreover, modified LDL elicits adaptive immune regulation, leading to substantial proliferation of inflammatory cells and the formation of thin fibrous caps, which profoundly influence atherosclerosis progression.^383–385^ These findings highlight oxidized LDL and other modified LDL forms as antigens that drive immune responses in atherosclerosis.

When the cholesterol derived from lipoproteins absorbed by macrophages exceeds the amount excreted, intracellular free cholesterol is converted into cholesterol esters. These cholesterol esters accumulate in LDs, resulting in the foam cell morphology observed by early pathologists.^386^ This leads to increased LD content within foam cells, further causing apoptosis, secondary necrosis, and inflammatory responses.^387^ Notably, strategies aimed at attenuating cholesterol storage, enhancing cholesterol efflux pathways and promoting LD breakdown may alleviate plaque inflammation and potentially regress plaques.^88,388,389^ HDL mitigates atherosclerotic lesions by promoting cholesterol removal from macrophage foam cells and preventing LDL lipid peroxidation.^371^ Furthermore, HDL protects against atherogenesis by enhancing the activation of endothelial NO synthase and inducing NO to improve endothelial barrier integrity, vasodilation and the number of endothelial progenitor cells.^390,391^ For LDs, macrophage foam cells promote LD breakdown through autophagy to maintain cellular lipid homeostasis, which may also represent a novel strategy for treating atherosclerosis.^389^

Genetic variations can affect lipid synthesis, transport, and degradation, thus having a profound impact on cardiovascular health. Taking the polymorphic protein ApoE as an example, several rare *ApoE* gene variations are found in different types of lipid abnormalities, including familial dysbetalipoproteinaemia, familial combined hyperlipidemia, lipoprotein nephropathy, and true autosomal dominant hypercholesterolemia (ADH). The APOE-p. The Leu167del variant has been identified as a pathogenic molecular component in two different ADH families.^392^ CETP is another protein closely associated with lipid metabolism. CETP variant carriers have higher plasma HDL cholesterol levels, lower plasma LDL-C levels, and a lower risk of experiencing atherosclerotic CVD events during follow-up. This finding suggests that common genetic variations in the *CETP* gene region are related to cardiovascular recovery during aging.^393^

### Lipid metabolism in aging

Aging is characterized by systemic chronic inflammation accompanied by cellular aging, immunosenescence, organ dysfunction, and age-related diseases.^394^ In recent decades, numerous genetic pathways regulating lifespan have been identified. Notably, many of these regulatory pathways are associated with lipid metabolism. Lipid metabolic enzymes undergo significant changes during the aging process and are regulated by various longevity pathways. Lipids also function as signaling molecules that actively modulate lifespan and healthspan. For example, compared with shorter-lived metazoan species, the longest-lived metazoan species, the mollusk *Arctica islandica*, has higher levels of MUFAs in its cell and mitochondrial membrane phospholipids.^395^ In the Leiden longevity study, plasma lipidomic analysis of middle-aged offspring of nonagenarian siblings and their partners revealed that female offspring presented a lower PUFA/MUFA ratio, whereas male offspring presented no difference.^396^ Therefore, the associations between FAs and lifespan vary depending on tissue, sex, and species.

Moreover, in female mice, a lack of DGAT1 promotes longevity and leanness without decreasing food consumption. These mice also reduce cholesterol biosynthesis and are protected from age-related obesity and inflammation in white adipose tissue.^397^ In the nematode *Caenorhabditis elegans*, overexpression of ATGL or muscle-specific activation of protein kinase A extends lifespan.^398,399^ Furthermore, ApoE, the most abundant brain-associated lipoprotein, has three main allelic variations: ε2, ε3, and ε4. The ε4 allele is correlated with an increased risk of AD, whereas the ε2 allele is linked to neuroprotection, anti-AD effects, and human longevity.^400^

With age, the efficiency of lipid metabolism typically decreases, leading to cellular dysfunction, energy metabolism disturbances, and membrane structure damage, which accelerate the aging process. However, genetic variations in lipid metabolism-related genes can significantly influence these processes. Studies have shown that genetic variations in lipid-lowering drug target genes (such as proprotein convertase subtilisin/kexin type 9 (*PCSK9), CETP, lipoprotein lipase (LPL), LDLR*, and *APOC3*) are associated with increased human lifespan.^401^ Furthermore, genetic variations in *APOL2* regulate the impact of long-term heavy drinking on epigenetic age acceleration, with the minor allele A of S9 16264 closely associated with increased epigenetic age acceleration and hippocampal mRNA expression.^402^ Additionally, genetic variations in the *LXRα* (*nuclear receptor subfamily 1 group H* member 3 (*NR1H3*)) gene are closely related to human lifespan, with a common haplotype of the *NR1H3* gene associated with prolonged lifespan, primarily due to reduced infectious disease mortality.^403^ Molecular characterization of longevity-promoting signaling pathways has revealed the mechanistic link between lipid metabolism and longevity. These lipid signaling pathways, mechanisms and their relationships with aging have been extensively discussed.^404^

### Metabolic disorders

Metabolic disorders are a group of diseases caused by abnormal metabolic processes within the body, including obesity, metabolic syndrome, and fatty liver disease. Fatty liver disease is driven primarily by excessive lipid accumulation.^405^ Metabolic dysfunction-associated steatotic liver disease (MASLD), previously known as nonalcoholic fatty liver disease (NAFLD), affects approximately 25% of the global population.^405,406^ Additionally, the complex pathogenesis of MASLD involves various metabolic disturbances, including lipid metabolism disorders, insulin resistance, obesity, and metabolic syndrome, which promote inflammation in the liver and increase the risk of cirrhosis and hepatocellular carcinoma.^407,408^ Therefore, developing effective therapies for metabolic disorders has become a focal point of research.

High-fat diets can induce MASLD in obese and diabetic rats, altering BA composition due to liver damage and slightly increasing the total BA pool, particularly with elevated levels of DCA and taurodeoxycholic acid (TDCA).^409,410^ However, serum hyodeoxycholic acid (HDCA) levels are negatively correlated with NAFLD severity, and a decrease in HDCA is a key change observed in hypertensive NAFLD models.^411^ Dietary HDCA supplementation has been shown to improve diet-induced NAFLD in male wild-type mice by activating PPAR-α-dependent FAO in the liver.^411^ Moreover, UDCA regulates the expression of Trem-1 and Trem-2 in primary cultured mouse Kupffer cells and suppresses inflammatory gene transcription through a TREM-2-dependent mechanism.^412^ This may serve as a potential approach to alleviate MASLD. Interestingly, in children with NAFLD undergoing oral glucose tolerance tests, most BA responses to glucose are blunted, with only glycine- and taurine-conjugated CDCA and hyocholic acid (HCA) changing in response, indicating that these secondary BAs may have potential as diagnostic and therapeutic targets for NAFLD.^413^ HDCA treatment has been shown to alleviate NAFLD in various mouse models by inhibiting FXR and upregulating hepatic cytochrome P450 family 7 subfamily B member 1 (CYP7B1).^414^ In addition, tauroursodeoxycholate (TUDCA) can inhibit FXR and FATP5 expression, reduce FA absorption and hepatic lipid accumulation, enhance gut barrier function, and improve the gut microbiota to alleviate NAFLD.^415^ Conversely, research suggests that upregulating FXR and bile salt export pumps can regulate BA metabolism, reducing the serum and liver fat contents in high-fat diet-fed mice and effectively alleviating NAFLD progression.^416^ These findings highlight the bidirectional regulatory role of FXR in NAFLD.

Increased free FA uptake, defective free FA oxidation, and decreased lipid export can all impair hepatic lipid metabolism.^417^ Specifically, palmitic acid can induce Kupffer cells to secrete TNF, thereby inhibiting FA metabolism and oxidative phosphorylation and ultimately promoting the development of fatty liver.^418^ Moreover, one study suggested that reducing plasma SFAs and MUFAs while increasing n-3 and n-6 PUFA intake may offer protection against severe NAFLD, which is likely mediated by lipid metabolism and inflammation.^419^ In terms of FABP, serum A-FABP levels are significantly elevated in NAFLD patients and are positively correlated with liver fat percentage.^420^ Knocking out or knocking down FATP5 reduces hepatocyte fatty acid uptake, reversing diet-induced hepatic steatosis in mice.^420^

LPL is one of the most important enzymes in lipid metabolism and is responsible for hydrolyzing triglycerides in lipoproteins to release free FAs for energy or storage in adipose tissue. Genetic mutations in the *LPL* gene can lead to reduced enzyme activity, resulting in the accumulation of triglycerides in the blood, thus increasing the risk of hypertriglyceridemia. For example, a report indicated that carriers of LPL heterozygous mutations or deletions may exhibit normal plasma lipid levels or develop familial chylomicronemia syndrome.^421^ Another enzyme that plays an important role in lipid metabolism is CETP. Variants of the *CETP* gene, such as *rs1800777*, are independently associated with the presence of fat degeneration and lobular inflammation in biopsy-confirmed NAFLD patients.^422^ The *PCSK9* gene is also crucial in lipid metabolism. PCSK9 regulates LDL cholesterol clearance by controlling the number of LDLRs on liver cells. *PCSK9* variants associated with lower LDL cholesterol levels are also related to higher circulating fasting blood glucose concentrations, weight, waist‒to-hip ratios, and an increased risk of T2DM.^423^

In summary, targeting lipid metabolism pathways (such as the PPAR signaling pathway and FAO), gut microbiota metabolites (such as secondary BAs), secondary BAs and their receptors (such as nuclear receptors such as FXR), and dietary and lifestyle interventions (such as the intake of n-3 and n-6 PUFAs) is a promising approach for the prevention or treatment of metabolic disorders.

### Application of lipid metabolic profiles in clinical research

Most diseases, such as autoimmune diseases, cancer, and obesity, are associated with abnormalities in lipid metabolism.^424–427^ Therefore, analyzing lipid metabolic profiles can reveal specific metabolic patterns that are closely related to the clinical manifestations of the disease. These patterns not only help in early diagnosis but also provide potential insights for personalized treatment. For example, differentiating between seronegative RA and psoriatic arthritis (PsA) is often challenging. By establishing serum metabolomics and lipidomic diagnostic models, it has been found that alanine, succinate, and phosphocreatine concentrations, along with lipid ratios, are valuable indicators for distinguishing between seronegative RA patients and PsA patients.^428^ Furthermore, metabolic markers such as acetate, creatine, glycine, and formate, along with lipid ratios such as L1/L6, can reliably predict, with high sensitivity and specificity, whether patients with arthritis or peripheral neuropathy have cancer, thus assisting in early cancer diagnosis.^426^

Abnormal lipid metabolism is also useful in tumor screening and stratification. For example, dysregulation of FAs and phospholipids, particularly alterations in glycerophospholipid metabolism, can distinguish obese individuals with CRC from those with obesity alone, potentially enabling more accurate CRC screening.^429^ These lipid metabolic changes provide biomarkers closely associated with disease onset and progression, offering critical support for early diagnosis and prognosis assessment. Lipid metabolic profiling not only aids in patient stratification but also provides important clues for personalized treatment strategies. Studies have shown that specific lipid metabolites can serve as potential biomarkers for predicting inflammation and treatment response. By quantitatively analyzing lipid metabolites in blood or tissue samples, patients can be accurately classified, enabling clinicians to tailor the most appropriate treatment plan. For example, plasma and fecal metabolomics have established a diagnostic model consisting of 17 plasma metabolites, particularly oleic acid and LCA, which can identify functional metabolites involved in the progression of adenoma to CRC and serve as early diagnostic biomarkers.^424^ As lipidomic technologies continue to advance, the potential of lipid metabolic profiling in clinical applications will become increasingly significant, particularly in the management of inflammatory diseases, cancer, and metabolic disorders, thus enabling more personalized and precise disease management.

## Targeting lipid metabolism in immune cells for disease therapy

**Fig. 7:**
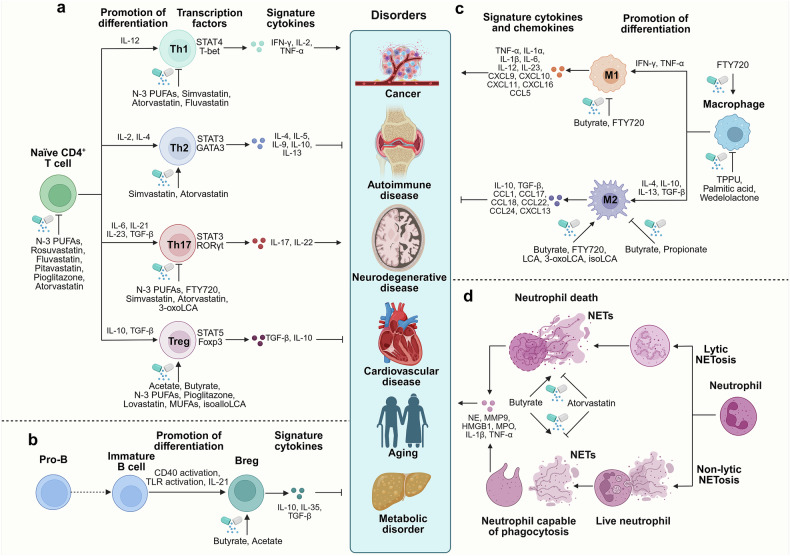
Drug-mediated immune cell differentiation in various disorders. **a** Drugs reprogram CD4⁺ T cell differentiation into Th1, Th2, Th17, and Treg subsets, thereby modulating immune responses across a range of diseases. **b** Drugs influence B cell maturation, antibody production, and Breg differentiation, thus regulating humoral immunity. **c** Drugs induce macrophage polarization into M1 or M2 phenotypes, shaping inflammatory responses and contributing to tissue homeostasis under diverse pathological conditions. **d** Drugs regulate neutrophil activation, NETosis (both lytic and non-lytic), and effector functions, offering therapeutic strategies for neutrophil-driven inflammation. Lipid metabolism-targeting drugs modulate cytokine and chemokine production *via* immune cell reprogramming, suggesting their potential in disease prevention and treatment. Created in BioRender

Given the crucial functions of lipid metabolism in regulating immune cells and human ailments, there is a growing focus on the utilization of lipid metabolism regulation for therapeutic purposes. In this context, potent approaches targeting lipid metabolism and signaling to treat related diseases have been outlined, as shown in Fig. 7 and Table 2, and we have further summarized the current clinical trials of drugs related to lipid metabolism (Table 3).

**Table 2:** Targeting lipid metabolism to regulate immune cells for disease treatment

Categories	Treatments	Animal models/patients	Diseases	Mechanisms of action	Refs.
FAs	Butyrate	Arthritic mice	RA	AhR^+^ Breg (+); GC B cell (-)	^437^
	Butyrate	Imiquimod-induced psoriasis mice	Psoriasis	Treg, IL-10 (+); IL-17, IL-6 (-)	^439^
	Propionate	MS patients; EAE mice	MS	Treg (+); Th1, Th17 (-)	^48,81^
	Acetate/Butyrate	NOD mice	T1DM	Treg (+); IL-21, B cell (-)	^308^
	Acetate	Colitis; Arthritis; Asthma mice	Inflammatory responses	Neutrophil, GPR43 (+)	^312^
	Propionate/Butyrate	Rat	Inflammation	Neutrophil, L-selectin, CINC-2αβ (+)	^61^
	Butyrate	DSS-induced murine colitis	IBD	Neutrophil, NET (-)	^438^
	N-3 PUFAs	NOD mice	T1DM	Treg, Th2 (+); Th1, Th17, IFN-γ, IL-17, IL-6, TNF-α (-)	^316^
	Palmitic acid	Obesity mice	Obesity	Macrophage, MD2-TLR4 (+)	^84^
	Oleic acid	MS patients	MS	Foxp^+^ Treg FAO-oxidative phosphorylation (+)	^79^
S1PR modulators	FTY720	Experimental autoimmune encephalomyelitis in LEW rats; SJL/J mice	MS	CD4^+^ T cell (-)	^443^
	FTY720	Patients with MS	MS	Th17, RORC2^+^, IL-17 (-)	^443^
	FTY720	MP4-induced experimental autoimmune encephalomyelitis	MS	T cell, B cell (-); TLO (+)	^444^
	FTY720	TNBS induced colitis mice	IBD	Th1 (-); Treg, IL-10, TGF-β, CTLA4 (+)	^522^
	FTY720	LDLR-deficient mice	Atherosclerosis	M1, TNF-α, IL-6, IL-12, IFN-γ (-); M2, T cell (+)	^158^
PPARs agonists	Pioglitazone	Human lupus and control PBMCs	SLE	CD4^+^ T cell (-)	^450^
	Bezafibrate	C57BL/6 N and BLAB/c mice with tumor	Cancer	FAO, Oxidative phosphorylation, Glycolysis, Cpt1, Bcl2 (+)	^454^
Statins	Simvastatin	PBMCs from SLE patients	SLE	Th17, IL-17, IL-21, RhoA-ROCK (-)	^433^
	Simvastatin	Relapsing remitting MS; MOG (35-55) induced mice	MS	Th1, Th17, IL-6, IL-12, RANTES, MIP-1β (-)	^459^
	Atorvastatin	Cecum ligation and puncture-induced lung injury mouse model	Sepsis	Neutrophil, ERK/NOX2, NETosis (-)	^457^
	Atorvastatin	Chronic and relapsing EAE	MS	Th2, IL-4, IL-5, IL-10, TGF-β (+); Th1, IL-2, IL-12, IFN-γ, TNF-α, MHC-II (-)	^456^
	Fluvastatin	EAM was induced in Lewis rats by immunization with myosin	Myocarditis	Th1, IFN-γ, IL-2 (-)	^462^
	Liposome-encapsulated pravastatin	Murine B16F10-melanoma	Cancer	M-CSF, IGF-II, IL-1α, IL-1β, Leptin, IL-6, TNF-α (-); MHC-I (+)	^464^
	Lovastatin, Simvastatin, Mevastatin	PBMC obtained from untreated or IFN beta-1-treated patients with relapsing-remitting MS	MS	MMP9, Th1/Th17 (-)	^458^
	Rosuvastatin, Fluvastatin, Pitavastatin	Cytotoxic CD4 T cells from acute coronary syndrome patients	Acute coronary syndrome	ERK (+); CD69, TRAIL (-)	^463^
sEH inhibitors	TPPU	LPS-induced inflammation	AD	β amyloid (-); Synaptic integrity (+)	^467^
	TPPU	Lung injury and pulmonary fibrosis mice models	Pulmonary diseases	EET (+); MAPK/NF-κB, P450 oxidases/she (-)	^468,469^
	TPPU	Human subjects with chronic knee pain; Osteoarthritis model	Osteoarthritis	8,9-DHET, 14,15-DHET (-)	^470^
	Wedelolactone	LPS-induced acute lung injury	Acute lung injury	GSK3β, NF-κB, Nrf2 (-)	^471^
	A20	Neuropathic pain in rat model induced by spared nerve injury	Neuropathic pain	/	^473^

“+” represents activation, while “-” represents suppression

**Table 3:** Clinical progress in drugs targeting lipid metabolism

Categories	Drug	Targets	Indications	Clinical trial number	Approval phase	Refs.
Statin	Rosuvastatin	HMG-CoA reductase	Spinal cord injuries; Osteoporosis	NCT03113994	Phase II	/
			Chronic periodontitis	NCT02985099	Phase II/III	/
			Prostate cancer metastatic	NCT04776889	Phase IV	/
			NAFLD	NCT03434613	Phase IV	^493^
			Atherosclerotic CVD; T2DM	NCT03403556	Phase IV	^494^
			T2DM	NCT00747149	Approved	/
			Metabolic syndrome; Dyslipidemia	NCT00240305	Approved	^523^
			Hypercholesterolemia	NCT00329160	Approved	^524^
			Acute ischemic stroke	NCT05884502	Approved	/
			Arteriosclerosis; Lipid disorder; Diabetes mellitus	NCT02305862	Approved	/
	Simvastatin	HMG-CoA reductase	Celiac disease	NCT03011931	Early Phase I	/
			Smith-lemli-opitz syndrome	NCT00064792	Phase II	^525^
			Spinal cord injuries; Osteoporosis	NCT02946424	Phase II	^526^
			Chronic hepatitis C	NCT03490097	Phase II/III	^527^
			Chronic periodontitis with diabetes mellitus	NCT03745300	Phase II/III	/
			Noonan syndrome	NCT02713945	Phase III	/
			Intestinal neoplasm	NCT00994903	Phase III	^528^
			Serious mental illness; Schizophrenia; Schizoaffective disorder	NCT02188121	Phase IV	/
			Polycystic ovary syndrome	NCT00365638	/	^529^
			Obese	NCT01700530	/	^488^
			Metabolic syndrome	NCT00819403	Approved	/
			Dyslipidemia; Atherosclerosis	NCT00712049	Approved	/
			Hyperlipidemia; Hypertension	NCT02511418	Approved	^530^
	Atorvastatin	HMG-CoA reductase	Malignant disease; Solid tumor; Acute myeloid leukemia	NCT03560882	Phase I	/
			AD	NCT00024531	Phase II	^531^
			Psoriasis	NCT02432040	Phase II	/
			IBD	NCT05561062	Phase II	/
			Asthma; COPD; Smoking	NCT00463827	Phase II	^532^
			Liver fibroses; Cirrhosis	NCT05028829	Phase II	/
			MS	NCT00094172	Phase II	^533^
			SLE	NCT00065806	Phase III	^489^
			Osteoporosis	NCT02342015	Phase IV	/
			Bronchiectasis	NCT01299181	Phase IV	^534^
			Dementia, mixed; Dementia, vascular; Dementia of Alzheimer type	NCT05586750	Phase IV	/
			Hypercholesterolemia; Dyslipidemias	NCT03611010	Approved	/
			Coronary artery disease; Atherosclerosis	NCT01013103	Approved	^535^
			T2DM	NCT03018444	Approved	/
			Myocardial infarction; Myocardial ischemia	NCT00967434	Approved	^536^
	Fluvastatin	HMG-CoA reductase	Breast cancer	NCT00416403	Phase II	^490^
			Antiphospholipid syndrome	NCT00674297	Phase II	^491^
			Mixed dyslipidemia; Hypercholesterolemia	NCT00136799	Approved	/
			Coronary disease; Myocardial infarction	NCT00171275	Approved	^537^
			Osteoporosis	NCT00489424	Phase IV	/
			Lipid metabolism disorders	NCT01551173	Approved	/
	Pitavastatin	HMG-CoA reductase	Glioblastoma multiforme, adult; Recurrent glioblastoma	NCT05977738	Early Phase I	/
			Osteoporosis, osteopenia; Hypercholesterolemia; Menopausal; Osteoporosis	NCT06359353	Phase IV	/
			MS	NCT00444717	Approved	^538^
			Atherosclerosis; Angina	NCT02545231	Approved	^539^
			Diabetes mellitus; Glucose intolerance	NCT00301392	Approved	/
			Hypercholesterolemia; Dyslipidemia	NCT00325780	Approved	^540^
			NAFLD	NCT02290106	Approved	^541^
PPAR agonist	Pemafibrate	PPAR-α	Diabetic retinopathy	NCT03345901	Phase III	/
	OMT-28	PPAR-α	Primary mitochondrial disease	NCT05972954	Phase II	/
	Fenofibrate	PPAR-α	Diabetic macular edema	NCT00683176	Phase II	/
			IBD	NCT05781698	Phase II	/
			NASH with hypertriglyceridemia	NCT02781584	Phase II	^503^
			Primary Biliary Cirrhosis	NCT02823353	Phase III	^501^
			Hypertension	NCT00872599	Phase IV	^502^
			Coronary heart disease; Hyperlipidemia	NCT00552747	Phase IV	/
			T2DM; CVDs	NCT05542147	/	^542^
			Dyslipidemia; Atherosclerosis; T2DM	NCT05365425	Approved	/
	Ciprofibrate	PPAR-α	Myocardial insulin sensitivity; Impaired glucose metabolism; Diastolic dysfunction	NCT03662984	Phase III	/
			Hypertension; Dyslipidemia	NCT00350038	Phase IV	/
	Elafibranor	PPAR-α/δ	Hepatic impairment; Liver disease	NCT03765671	Phase I	/
			Renal impairment; Renal insufficiency; Kidney diseases	NCT03844555	Phase I	/
			Nonalcoholic steatohepatitis	NCT03883607	Phase II	/
			Primary biliary cholangitis	NCT06383403	Phase III	/
			Primary biliary cirrhosis	NCT04526665	Phase III	/
	Pioglitazone Hydrochloride	PPAR-γ	Brain neoplasms, malignant; Brain neoplasms, benign; Malignant meningioma	NCT01151670	Phase I	/
			RA; Insulin resistance	NCT02535832	Phase I	/
			MS, relapsing-remitting	NCT00242177	Phase I	^543^
			PD	NCT01280123	Phase II	^544^
			Severe, refractory asthma; Airway inflammation; Airflow obstruction	NCT00994175	Phase II	^507^
			Breast cancer; Muscle fatigue	NCT05013255	Phase II	/
			Cancer of the pancreas	NCT01838317	Phase II	/
			Depressive disorder, major	NCT00671515	Phase II	/
			NAFLD; Steatohepatitis	NCT00013598	Phase II	/
			T2DM; Insulin Resistance	NCT05591235	Phase III	^505^
			T2DM	NCT05226897	Phase III	^509^
			Diabetes mellitus	NCT00672919	Phase IV	^545^
			Hypertension; Insulin resistance	NCT01472497	Phase IV	/
			NASH; Nonalcoholic fatty liver disease; T2DM	NCT00994682	Phase IV	^504^
			T2DM	NCT02231021	Phase IV	^508^
			Obesity	NCT00855010	/	/
			Psoriasis	NCT01133561	/	/
			T2DM	NCT04604223	Approved	/
			Metabolic syndrome	NCT00926341	Approved	^546^
	Rosiglitazone	PPAR-γ	AD	NCT00688207	Phase I	/
			Metabolic syndrome; Impaired glucose tolerance	NCT00370305	Phase II	^547^
			Prediabetes; Coronary artery disease; Insulin resistance	NCT01574820	Phase III	/
			T2DM	NCT00306176	Approved	^548^
N-3 FAs	N-3 acid ethyl esters	Lipid + Triglyceride	Human immunodeficiency virus	NCT00346697	Phase IV	^516^
			Metabolic syndrome; Hypertriglyceridemia	NCT00286234	Approved	^549^
sEH inhibitor	GSK2256294	sEH	Subarachnoid hemorrhage, aneurysmal; Delayed cerebral ischemia; Vasospasm, cerebral	NCT03318783	Phase I	^519^
			Pulmonary disease; Chronic obstructive	NCT01762774	/	^518^
			Obesity; Impaired glucose tolerance; Endocrine system diseases; Diabetes mellitus	NCT03486223	/	^517^
	EC5026	sEH	Healthy adults	NCT04228302, NCT04908995	/	^520^

### Targeting lipid metabolism or signaling alters immune cell function

Several inhibitors targeting enzymes involved in lipid metabolism pathways have been developed. Both genetic and chemical approaches have demonstrated the specificity and effectiveness of these inhibitors in modulating immune cell function. Statins, including rosuvastatin, simvastatin, atorvastatin, pitavastatin, and fluvastatin, are among the most widely used drugs for managing dyslipidemia and reducing cardiovascular events. They work by inhibiting HMG-CoA reductase, the rate-limiting enzyme in cholesterol biosynthesis, leading to decreased levels of LDL-C and total cholesterol in the blood. As HMG-CoA reductase inhibitors, statins inhibit MCP-1-induced monocyte chemotaxis and reduce the secretion of MCP-1 and matrix metalloproteinase-9 (MMP9) from cultured macrophages, thereby preventing plaque destabilization and rupture.^430^ Atorvastatin, fluvastatin, and lovastatin have been reported to upregulate CD36 expression by disrupting cytoskeleton organization through inactivation of Rho GTPases in human monocytes.^194^ Moreover, combination therapy with lovastatin and paclitaxel increases CD8^+^ T cell infiltration, increases their tumor-killing capacity, and improves their in vivo efficacy.^431^ Another study illustrated the influence of lovastatin on Tregs and revealed that lovastatin increases migration and cell count in inflamed tissue, enhancing the suppressive effects through Foxp3 induction. Notably, statins indirectly affect Treg differentiation by promoting tolerogenic properties in DCs.^432^

Statins such as simvastatin interfere with Ras homolog family member A (RhoA) activation by inhibiting HMG-CoA reductase, thereby reducing Rho-associated coiled-coil kinase (ROCK) activity in Th17 cells. Concurrently, they decrease the production of IL-17 and IL-21 in purified SLE T cells or Th17 cells.^433^

Lipid agonists that activate PPARs influence immune cell fate. For example, the activation of PPARγ enhances Treg responses through the upregulation of CD36/CPT1-mediated fatty acid oxidation and subsequent N-glycan branching of TβRII/IL-2Rα.^434^ Similarly, pioglitazone, another PPAR-γ ligand, increases Treg accumulation in visceral adipose tissue (VAT), whereas treatment of obese mice with a small-molecule PPARγ agonist limits Th17-driven pathology and restores responsiveness to targeted anti-Th2 biologics.^435,436^

### Targeting lipid metabolism or signaling for disease therapeutics

FAs are crucial for both health maintenance and disease development, and FA supplementation has emerged as an effective treatment method. Butyrate supplementation has been shown to ameliorate RA progression in a Breg-dependent manner by increasing 5-hydroxyindole-3-acetic acid levels, activating the aryl hydrocarbon receptor, and increasing IL-10 expression.^437^ Additionally, the dietary inclusion of butyrate and acetate impacts the differentiation and function of T and B cells, contributing to intestinal equilibrium and reducing IL-21 levels, which aids in T1DM prevention.^308^ Like butyrate treatment for diabetes, supplementation with n-3 PUFAs can alleviate diabetes pathogenesis by influencing T cell and macrophage proliferation and differentiation, thereby suppressing IL-6, IL-17, and IFN-γ production.^316^ Moreover, dietary supplementation with butyrate also alleviates mucosal inflammation in IBD by inhibiting the migration and release of neutrophil NETs.^438^ Notably, compared with systemic application, topical administration of sodium butyrate modulates IL-10 and Foxp3 expression, diminishing imiquimod-induced skin thickening, scaling, and inflammation.^439^

FAs also impact the response to cancer immunotherapy. The immunomodulatory properties of SCFAs, particularly butyrate, may be applicable to tumor-specific cytotoxic T lymphocytes and CAR-T cells. Studies have demonstrated that butyrate enhances the antitumor immune response of cytotoxic CD8^+^ T cells both in vivo and in vitro through IL-12 signaling.^42^ Furthermore, dietary intake of n-3 PUFAs has been demonstrated to modulate immune responses and delay tumor growth.^440^ Further exploration is warranted to clarify the precise role of SCFAs in cellular metabolism and their influence on anti-inflammatory reactions.

S1P, a crucial signaling molecule, regulates cellular signaling and immune cell transport. Receptor modulators of S1P play pivotal roles in various physiological and pathological processes.^441,442^ FTY720, an S1PR modulator, is used to treat MS by reducing the population of circulating naive and central T cells, notably Th17 cells, and preventing the formation of B-cell clusters in the central nervous system.^443–445^ FTY720 administration also slows atherosclerosis progression in LDLR-deficient mice by suppressing TNF-*α*, IL-6, and IL-12 expression and enhancing T-cell and macrophage activity.^158^ Moreover, the ability of FTY720 to heal colitis lesions is linked to its ability to inhibit the effects of S1P on lymphocytes and endothelial cells; increase Foxp3, TGF-*β*, IL-10, and cytotoxic T-lymphocyte antigen-4 (CTLA-4) expression; and enhance Treg cell function.^446^ With the successful development of S1PR modulators, the influence of S1P on diseases is expected to gain further prominence.

PPARs are ligand-activated receptors in the nuclear hormone receptor family that control various intracellular metabolic processes. Among them, PPAR-γ agonists are under active development and investigation because of their involvement in immune responses and disease progression. Pioglitazone, a PPAR-γ agonist, alleviates joint swelling, diminishes skin lesions, and benefits patients with PsA, albeit with potential side effects such as weight gain and peripheral edema.^447^ Moreover, pioglitazone significantly improves insulin resistance; reduces the levels of ROS, TNF-*α*, and IL-1*β*; and ameliorates RA symptoms.^448,449^

In SLE patients, pioglitazone selectively modulates CD4^+^ T cell proliferation and activation, indicating its potential as a therapeutic option for this disease.^450^ In EAE, pioglitazone has therapeutic effects by inhibiting T cell activation and expansion in the brain, offering a potential foundation for MS treatment.^451^

Furthermore, pioglitazone treatment reproduced the antileukemic effects of selenium supplementation by inducing selenium-dependent PPARγ activation in chronic myeloid leukemia. This activation leads to Stat5 downregulation, impairing leukemic stem cell maintenance and thereby suppressing disease progression.^452^ The combined use of pioglitazone and rofecoxib has demonstrated efficacy in slowing the progression of melanoma and soft tissue sarcoma through the upregulation of proapoptotic cells, showing promise as a potential therapeutic option for malignant vascular tumors.^453^ Combining PPAR-γ coactivator 1-alpha complex agonists with PD-1-blocking monoclonal antibodies has been shown to enhance functional cytotoxic T lymphocyte populations, bolstering antitumor resistance, as supported by previous studies.^454^

Statins, specifically HMG-CoA reductase inhibitors, are renowned for hindering cholesterol synthesis. They effectively reduce LDL-C and triglyceride levels while curbing oxidative and inflammatory reactions.^455^ Atorvastatin effectively ameliorates EAE in mice, primarily by reducing central nervous system inflammatory infiltration *via* the modulation of CD4^+^ T cell proliferation and differentiation.^456^ In addition to its effects on MS, atorvastatin effectively suppresses NET formation, significantly attenuates pulmonary injury in septic mice, and reduces systemic inflammation. Its mechanism of inhibiting NET formation may involve the ERK/NADPH oxidase 2 (NOX2) pathway.^457^ Like atorvastatin, simvastatin slows MS progression by promoting Th2 cell differentiation while impeding Th1/Th17 cell function and the expression of IL-17, IL-22, and IL-21.^458,459^ Moreover, simvastatin also reduces lipid raft abundance by depleting cholesterol, thereby alleviating atherosclerosis and cancer.^460,461^

Other statins, such as fluvastatin, can reduce IFN-γ expression in the myocardium by modulating CD4^+^ T cell differentiation.^462^ Additionally, rosuvastatin, fluvastatin, and pitavastatin inhibit the enrichment of CD4^+^ T cells and endothelial cell apoptosis by decreasing CD69 and TNF-related apoptosis-inducing ligand expression on T cells, leading to improved plaque stability and the alleviation of acute coronary syndrome.^463^ These findings suggest that different types of statins have similar effects on disease treatment.

Furthermore, statins exhibit antitumor properties. Liposomal pravastatin, for example, exerts its antitumor effects by upregulating MHC-I and suppressing proinflammatory factors and angiogenesis within tumor tissues. These findings underscore the therapeutic potential of lipid metabolism-related drugs in managing certain cancers.^464^

These preclinical studies lay the foundation for the clinical application of these drugs, highlighting the critical role of lipid metabolism regulation in the treatment of various diseases and driving the development and clinical translation of related drugs.

### Targeting lipid metabolism by preventing the degradation of proresolving lipids

Soluble epoxide hydrolase (sEH) is an important enzyme involved in the metabolism of bioactive FAs, specifically epoxy FAs within the arachidonic acid signaling pathway. By converting these epoxy FAs into diols, sEH alters their bioactivity, typically promoting proinflammatory effects.^465^ Inhibiting sEH, therefore, holds great therapeutic potential by increasing the levels of epoxy eicosatrienoic acids (EETs) and suppressing inflammatory pathways,^466^ making sEH a promising therapeutic target for immune-mediated diseases.

Specific small molecule sEH inhibitors have demonstrated significant anti-inflammatory and protective effects across various disease models. For example, TPPU (1-trifluoromethoxyphenyl-3-(1-propionylpiperidine-4-yl) urea, an sEH inhibitor) protects wild-type mice from LPS-induced systemic inflammation.^467^ In lung injury models, TPPU alleviates lung damage by increasing EET levels in mice and suppressing alveolar macrophage activity *via* the MAPK/NF-κB pathway.^468^ Furthermore, TPPU reduces age-related pulmonary fibrosis, highlighting its therapeutic potential for pulmonary diseases.^469^ In osteoarthritis models, acute and chronic systemic administration of TPPU reverses established pain behaviors and decreases circulating levels of 8,9-dihydroxy-eicosatrienoic acid (8,9-DHET) and 14,15-DHET, demonstrating its analgesic properties.^470^

Another sEH inhibitor, wedelolactone, targets sEH by interacting with the amino acids Phe362 and Gln384, thereby inhibiting the NF-κB and nuclear factor erythroid factor 2 (Nrf2) signaling pathways. This results in macrophage inactivation, reduced inflammation, and oxidative stress.^471^ Additionally, EC5026 is advancing toward clinical application as a novel dual-function therapeutic, demonstrating efficacy in neuropathic pain management while offering a nonaddictive alternative to opioids.^472^ Interestingly, compound A20, identified through sEH-targeted screening, alleviates neuropathic pain in rats in a dose-dependent manner, outperforming gabapentin and the sEH inhibitor EC5026.^473^ These studies collectively suggest that sEH inhibitors hold broad therapeutic potential for managing inflammation, pain, fibrosis, and inflammation-related diseases.

In parallel, specialized proresolving mediators (SPMs) have emerged as key players in the resolution of inflammation. These bioactive lipids, such as resolvins, protectins, and maresins, are synthesized during the resolution phase of inflammation and help to clear cellular debris, promote tissue repair, and counteract the activation of immune cells involved in chronic inflammation.^32,474^ SPMs exert anti-inflammatory effects by interacting with specific receptors on immune cells to halt excessive inflammation and promote the healing of damaged tissues.^475^ For example, SPMs, including protectins, maresins, and D-series resolvins, play crucial roles in enhancing the ability of macrophages to utilize endogenous prostaglandin E receptor 4 (EP4) in conjunction with Gi-type G-proteins. This coupling allows EP4 to shift from an antiphagocytic function to one that actively promotes phagocytosis, a core mechanism in the resolution of inflammation.^476^ In addition, exogenous supplementation with specific SPMs, such as 17-hydroxydocosahexaenoic acid (17-HDHA, an intermediate in the production of RvD1) and RvD1 itself, has been shown to increase the production of human B cell antibodies, including IgM and IgG. Furthermore, studies using in vitro B cells from donors (stimulated to produce IgE) and B cells from asthma patients have demonstrated that both RvD1 and 17-HDHA have profound inhibitory effects on the number of B cells that undergo class switching to produce IgE. Moreover, lipoxin B4 (LXB4) suppresses IL-10 production by Bregs and inhibits the maturation of resting memory B cells into antibody-secreting cells.^475^

In addition to these effects on B cells, several SPMs, including RvD1, RvD2, Maresin 1 (MaR1), RvD3, LXA4, and LXB4, have been shown to block Th1 and Th17 cell production of proinflammatory cytokines such as IFN-γ and TNF-α.^477^ RvD1, RvD2, and MaR1, in particular, are capable of inhibiting the differentiation of human peripheral blood monocytes into Th1 and Th17 cells in vitro while simultaneously enhancing their ability to differentiate into Tregs.^478^ Notably, MaR1 enhances the ability of Tregs to suppress the production of inflammatory cytokines by type 2 innate lymphoid cells, further contributing to the regulation of inflammation. Additionally, RvE1 has been shown to inhibit the differentiation of mouse spleen T cells into Th17 cells in vitro.^479^

SPMs can effectively modulate the infiltration of neutrophils, the production of cytokines and chemokines, and the clearance of apoptotic neutrophils by macrophages, thereby promoting tissue restoration and balance.^480^ Specifically, RvE1 attenuates intestinal inflammation by promoting neutrophil spherocytosis and macrophage-derived IL-10 secretion.^481^ In addition, RvD1 can inhibit both neutrophil recruitment and aggregation during the initial inflammatory phase.^481^ Furthermore, RvD1 suppresses the infiltration of neutrophils, which is associated with the downregulation of miR-21 and miR-155 and the expression of actin polymerization and adhesion molecules.^482^

Given that both sEH inhibitors and SPMs play complementary roles in regulating immune responses, combining these approaches can result in synergistic effects. By increasing the levels of EETs through sEH inhibition and promoting the resolution of inflammation with SPMs, a more comprehensive strategy can be developed to manage chronic inflammatory diseases.

### FDA-approved drugs and clinical trials

Lipid metabolism is central to various diseases, such as dyslipidemia, atherosclerosis, MS, osteoporosis, obesity, and NAFLD. Several FDA-approved drugs have been developed to modulate lipid metabolism, improve lipid profiles, and reduce the risk of CVDs. These drugs act through various mechanisms, such as inhibiting cholesterol synthesis, enhancing lipid transport, or modifying lipid-related gene expression. In this section, we review the therapeutic strategies employed by several FDA-approved drugs for lipid metabolism and discuss their efficacy, safety, tolerability, side effects, and outcomes, with an emphasis on their clinical impact.

#### Statins

Statins are primarily used to treat CVDs (such as high cholesterol, atherosclerosis, and coronary heart disease), diabetes, and metabolism-related disorders (such as metabolic syndrome). As shown in Table 3, rosuvastatin, simvastatin, and atorvastatin have been approved for the treatment of high cholesterol, atherosclerosis, and diabetes, with significant lipid-lowering effects that effectively reduce LDL levels and decrease the occurrence of cardiovascular events. Their safety is relatively high, and they are well tolerated; however, long-term use may lead to side effects such as muscle pain, liver function abnormalities, and indigestion, particularly at high doses. Simvastatin, when used in combination with certain other drugs, may increase the risk of side effects.^483–485^ Fluvastatin and pitavastatin are primarily used to treat mixed dyslipidemia, coronary heart disease, atherosclerosis, and diabetes. The efficacy of these drugs is similar to that of the aforementioned drugs, which significantly improve blood lipid levels and reduce cardiovascular events. The side effects mainly include muscle pain and elevated liver enzymes, but these drugs have a relatively high safety profile and good tolerance.^483–485^ Overall, statins have unique advantages. They are supported by extensive data from numerous large-scale randomized controlled trials, demonstrating their safety and efficacy. Additionally, their low cost alleviates the financial burden for patients requiring long-term medication.^486^ These findings lay the foundation for the successful market entry of statins.

In addition to these approved indications, these drugs are also undergoing clinical trials to explore their potential in treating other diseases. For example, in a phase II clinical trial (NCT00064792), simvastatin was found to be relatively safe in treating Smith–Lemli–Opitz syndrome (SLOS) patients, improving the ratio of serum dehydrocholesterol to total sterols and significantly alleviating irritability symptoms in mild to classic SLOS patients.^487^ Additionally, when combined with exercise training (NCT01700530), simvastatin alleviates the increase in cardiopulmonary function and mitochondrial content in the skeletal muscles of overweight or obese patients at risk of metabolic syndrome.^488^ A phase III clinical trial (NCT00065806) revealed that atorvastatin’s potential for preventing atherosclerosis in pediatric lupus erythematosus patients might be associated with underlying vitamin D deficiency.^489^ Another statin, fluvastatin (NCT00416403), has measurable biological effects by reducing tumor proliferation and increasing apoptosis in breast cancer cells, with the effect being more pronounced in high-grade tumors.^490^ Moreover, fluvastatin (NCT00674297) reversibly reduces proinflammatory and prothrombotic biomarkers.^491^

Regarding combination therapies, clinical trials have shown that the combination of rosuvastatin and ezetimibe reduces the number of aging CD8^+^ T cells while increasing the proportions of naive CD8^+^ T cells and memory CD8^+^ T cells. This change may help alleviate β-cell failure in T2DM patients, although the specific mechanisms involved remain unclear.^492^ Furthermore, a phase IV clinical trial (NCT03434613) revealed that the combination of rosuvastatin and ezetimibe significantly reduces liver fat content in NAFLD patients, and this combination therapy has been proven to be safe.^493^ Another phase IV clinical trial (NCT03403556) indicated that, compared with high-dose rosuvastatin monotherapy, moderate-dose rosuvastatin combined with ezetimibe provides superior therapeutic effects in high atherosclerotic CVD risk patients with T2DM.^494^ While the efficacy and safety of these drugs in new indications still need further verification, they show broad potential for application in cancer, neurological diseases (such as AD and MS), osteoporosis, and liver diseases.

#### PPARs

The currently approved PPAR agonists include fenofibrate, pioglitazone hydrochloride, and rosiglitazone, which are primarily used to treat metabolic syndrome, CVD, and diabetes and have shown significant efficacy in clinical studies. Pemafibrate and fenofibrate (PPAR-α agonists) have been approved for the treatment of dyslipidemia, atherosclerosis, and T2DM, effectively lowering blood lipids and improving metabolic conditions in individuals with diabetes. The side effects of these drugs are generally mild, mainly gastrointestinal discomfort, and most patients tolerate them well.^495–497^ Pemafibrate is currently being explored for its potential in treating diabetic retinopathy, atrial fibrillation, and primary mitochondrial diseases. Fenofibrate is also undergoing clinical trials for its effects on diabetic macular edema, IBD, hypertension, coronary artery disease, and breast cancer.

Pioglitazone hydrochloride and rosiglitazone (PPAR-γ agonists) have been approved for the treatment of T2DM and metabolic syndrome, effectively controlling blood glucose by improving insulin sensitivity and helping to reduce CVD risk. However, their side effects mainly include weight gain, edema, and potential heart failure risk, and they tend to have poorer tolerance, requiring careful monitoring during use.^498–500^ Additionally, pioglitazone hydrochloride is being studied for its effects on brain tumors, RA, MS, PD, and certain cancers. Rosiglitazone is currently being investigated for its potential in treating metabolic syndrome, prediabetes, and pulmonary inflammation.

Additionally, in a phase III clinical trial (NCT02823353), fenofibrate demonstrated good efficacy and tolerability in treatment-naive patients with primary biliary cholangitis.^501^ Moreover, in a phase IV clinical trial (NCT00872599), fenofibrate effectively reduced blood pressure, heart rate, and renal vascular contraction in salt-sensitive volunteers but had no significant effect on salt-resistant individuals.^502^ In patients with hypertriglyceridemia-associated NASH receiving cilofexor and firsocostat therapy, fenofibrate effectively mitigates triglyceride increases linked to ACC inhibition (NCT02781584).^503^

Furthermore, long-term use of pioglitazone is safe and effective for patients with prediabetes, T2DM, and NASH (NCT00994682).^504^ Interestingly, in T2DM patients, the fixed combination of metformin and pioglitazone significantly improved insulin resistance compared with metformin monotherapy (NCT05591235).^505^ However, pioglitazone is unlikely to alter the progression of early PD (NCT01280123), and further research in Parkinson’s disease patients is not recommended.^506^ Moreover, as 14% of subjects experience severe adverse reactions during pioglitazone treatment for severe asthma (NCT00994175), clinical trials of pioglitazone in severe asthma patients are also not advised.^507^ Pioglitazone plays a crucial role in triple-combination therapies. Clinical trials (NCT02231021) have shown that combining metformin, alogliptin, and pioglitazone is a valuable option for early-stage, poorly controlled T2DM patients.^508^ Additionally, adding pioglitazone to a combination of dapagliflozin and metformin has demonstrated good tolerability and significant improvements in glycated hemoglobin and glycemic levels in T2DM patients (NCT05101135, NCT05226897).^509,510^

As shown in Table 3, several clinical trials are investigating the therapeutic potential of PPAR-α and PPAR-δ agonists in various diseases progressing through different phases of development. In phase I clinical trials, elafibranor (a dual PPAR-α/PPAR-δ agonist) is being evaluated for its effects on liver and kidney diseases (NCT03765671, NCT03844555). In phase II, elafibranor is being tested for its efficacy in treating NAFLD (NCT03883607).^511^ Furthermore, in phase III trials, elafibranor is being investigated for the treatment of primary biliary cholangitis and primary biliary cirrhosis (NCT06383403, NCT04526665). The adverse events reported in these studies include abdominal pain, diarrhea, nausea, and vomiting.^512,513^ These studies highlight the potential of elafibranor as a promising therapeutic agent for various liver-related conditions.

In parallel, other PPAR-α agonists are also under investigation in clinical trials. OMT-28, a selective PPAR-α agonist, is currently being studied for its effects on atrial fibrillation and primary mitochondrial diseases (NCT05972954). Additionally, ciprofibrate, another PPAR-α agonist, is being evaluated for its impact on myocardial insulin sensitivity and cardiac and hepatic metabolism (NCT03662984).^514^ These trials suggest that PPAR-α activation may offer therapeutic benefits in a range of cardiovascular and metabolic disorders.

#### N-3 FAs

N-3 acid ethyl esters (n-3 FAs) have been clinically approved for the treatment of hypertriglyceridemia and metabolic syndrome. It significantly lowers triglyceride levels, improves lipid profiles, and reduces the risk of CVD. In clinical use, n-3 acid ethyl esters are generally considered safe, with mild side effects being the most common, including gastrointestinal discomfort such as nausea, diarrhea, and a fishy aftertaste.^515^

In addition to the approved indications, n-3 acid ethyl esters are being studied in several clinical trials to explore their potential in other diseases. For example, in a phase IV clinical trial (NCT00346697), the effects of n-3 acid ethyl esters on immune function in HIV patients are being investigated, and the results revealed that n-3 acid ethyl esters can decrease the concentrations of triglycerides, IL-6 and TNF-α in patients with well-controlled HIV infection and hypertriglyceridemia.^516^ The findings from these studies will help expand the clinical applications of N acid ethyl esters, particularly in the management of chronic inflammation and CVD.

#### sEH

While GSK2256294 and EC5026 have progressed to clinical studies as sEH inhibitors, TPPU remains untested in clinical trials. GSK2256294 has been studied primarily for the treatment of subarachnoid hemorrhage, aneurysms, cerebral vasospasm, and chronic obstructive pulmonary disease (COPD) (NCT03318783, NCT01762774). GSK2256294 has the potential to improve vascular health, reduce cerebral ischemia, and alleviate pulmonary inflammation. However, a phase II clinical trial (NCT03486223) failed to demonstrate its efficacy in treating impaired glucose tolerance, obesity and endocrine system diseases, even though it can inhibit sEH activity in plasma, muscle, and adipose tissue and reduce F2-isoprostanes, a marker of oxidative stress.^517^ The side effects of GSK2256294 are generally mild and include nausea, headache, and gastrointestinal discomfort. Most patients tolerate it well, although monitoring liver function and blood glucose is advised.^518,519^ For EC5026, a single-ascending dose study (NCT04228302) and a fed-fasted study (NCT04908995) demonstrated good tolerability, with no adverse reactions associated with EC5026 observed during treatment.^520^

In summary, several drugs have successfully transitioned from preclinical animal studies to clinical trials, demonstrating significant therapeutic effects. For example, n-3 FAs (such as vascepa) have been shown in preclinical studies to reduce triglycerides, exert anti-inflammatory effects, and improve atherosclerosis, with this mechanism being validated in clinical trials. Vascepa significantly reduced cardiovascular event risk in patients with high triglyceride levels. The PPAR agonist pioglitazone has been demonstrated in clinical trials to improve lipid and glucose metabolism, alleviate NAFLD and metabolic syndrome symptoms, and improve insulin sensitivity while lowering triglyceride levels. Additionally, statins (such as atorvastatin) have been proven in large-scale clinical trials to significantly reduce CVD risk by lowering LDL-C and cholesterol, especially in patients with low cholesterol. The successful translation of these drugs underscores the critical role of basic research in advancing clinical treatment, highlights the importance of lipid metabolism in various diseases, and reinforces the close link between preclinical research and clinical application, providing valuable insights for future drug development and therapeutic strategy innovations.

## Conclusions and future directions

Lipid metabolism and signaling within immune cells constitute a fundamental regulatory axis that orchestrates immune homeostasis, significantly influencing physiological responses and disease pathogenesis. Although significant progress has been made in uncovering lipid-mediated immunoregulatory mechanisms, translating these findings into clinically effective therapies remains challenging owing to inherent biological and technical complexity. The intrinsic complexity of lipid networks, comprising thousands of molecular species with precise spatiotemporal dynamics, presents substantial obstacles. Therefore, bridging the gap from bench insights to bedside applications will require innovative and interdisciplinary approaches.

A critical translational challenge lies in the context-dependent dual roles of lipid mediators, which frequently exhibit opposing immunomodulatory effects depending on the cellular microenvironment or pathological progression. This functional pleiotropy complicates therapeutic targeting, as systemic modulation risks impairing protective immune surveillance while suppressing deleterious inflammation. The compartmentalization of lipid metabolism further increases therapeutic unpredictability, as identical lipid species may exert distinct biological effects depending on their subcellular localization, incorporation into specific membrane domains, or biosynthetic origins. For example, mitochondrial cardiolipin derivatives and plasma membrane-derived sphingolipids engage separate signaling pathways despite sharing metabolic precursors, underscoring the importance of spatially targeted therapeutic strategies.

Current lipidomic methodologies remain insufficient for comprehensive pathway elucidation, particularly regarding the identification of low-abundance signaling lipids or the mapping of dynamic redistribution across subcellular compartments. Critical gaps persist in standardized protocols for lipid extraction, quantification, and data normalization, undermining cross-study reproducibility and biomarker validation efforts. Additionally, rapid lipidomic remodeling during immune activation frequently exceeds the temporal resolution capabilities of conventional analytical platforms, while the absence of harmonized reference databases impedes systematic comparisons across experimental models and clinical cohorts. These limitations contribute to fragmented insights into lipid‒immune crosstalk, delaying the identification of clinically relevant metabolic checkpoints.

Translational efforts also face substantial pharmacological hurdles, as targeting evolutionarily conserved lipid metabolic enzymes frequently triggers compensatory pathways or unintended metabolic disruptions in nontarget tissues. Given the widespread nature of lipid pathways across various cell types and organs, achieving immune cell-specific modulation remains particularly difficult, necessitating the development of novel strategies, such as metabolic priming agents or targeted drug delivery systems. Drug delivery complexities are further exacerbated by physicochemical barriers, particularly for compounds targeting organelle-specific lipid pools, where biological membrane permeability and intracellular pH gradients restrict effective biodistribution. Emerging nanocarrier systems, such as those incorporating pH-responsive lipid bilayers or organelle-targeting peptides, may partially overcome these barriers, but their long-term biocompatibility and immune specificity require further investigation.

Another significant revelation is the complex interaction between host lipid metabolism and microbiome-derived metabolites, adding further intricacy to therapeutic strategies. Gut microbiota-generated SCFAs and BA derivatives continuously reshape hepatic lipid synthesis and systemic immunometabolic circuits, contributing to interindividual variability that complicates treatment standardization. Currently, therapeutic strategies rarely integrate this host–microbiome axis, potentially accounting for the heterogeneous clinical responses observed in trials targeting lipid pathways. Future therapeutic approaches may require integrated modulation of microbial communities or dietary interventions to achieve consistent outcomes.

Technological advances offer transformative opportunities to overcome these barriers, particularly through the integration of single-cell lipidomics, spatial metabolomics, and artificial intelligence-driven network modeling. Next-generation analytical platforms capable of resolving lipid fluxes at subcellular resolution will be indispensable for mapping context-specific lipid interactomes, whereas machine learning algorithms could decode complex lipid‒protein interaction networks to identify novel druggable targets. Concurrent advancements in epigenetic editing and organelle-specific delivery systems can redefine therapeutic precision, enabling spatiotemporal control over lipid metabolic fluxes while minimizing off-target effects associated with conventional enzyme inhibitors.

Realizing personalized lipid immunotherapies demands extensive multiomics studies to elucidate the interplay among genetic polymorphisms, metabolic states, and environmental factors. Significant knowledge gaps persist regarding interactions between circadian metabolic rhythms, dietary lipid composition, and pharmacological efficacy, a nexus requiring dedicated investigation to optimize dosing schedules and nutritional cointerventions. Ethical considerations associated with long-term metabolic modulation must also be rigorously addressed, particularly regarding endocrine disruption, microbiome-mediated metabolic rebound, and equitable access to advanced lipid-targeting therapies.

Future clinical translation requires innovative therapeutic evaluation frameworks, transitioning from static biomarker assessments toward dynamic, real-time lipidome monitoring integrated with microbiome profiles and immune phenotypes. Adaptive clinical trial designs must account for individual metabolic heterogeneity and establish safety benchmarks regarding organ-specific drug distribution and potential off-target metabolic effects. Global consortia-driven standardization of lipidomic workflows and open-access databases will be critical for accelerating biomarker discovery and validating therapeutic mechanisms across diverse patient populations.

Ultimately, unlocking the full therapeutic potential of lipid immunomodulation requires unprecedented interdisciplinary collaboration, integrating lipid biochemistry, systems immunology, clinical pharmacology, and computational biology. Addressing these multidimensional challenges through innovative technologies and groundbreaking research could yield transformative therapeutic strategies that precisely recalibrate immune responses *via* lipid metabolic engineering. The coming decade will serve as a critical basis for determining whether lipid-targeted therapies can overcome current limitations and become cornerstone interventions in autoimmune disorders, cancer immunotherapy, and chronic inflammatory diseases.
